# Oncogenic accumulation of cysteine promotes cancer cell proliferation by regulating the translation of D-type cyclins

**DOI:** 10.1016/j.jbc.2024.107890

**Published:** 2024-10-15

**Authors:** Yumi Okano, Tomoaki Yamauchi, Runa Fukuzaki, Akito Tsuruta, Yuya Yoshida, Yuya Tsurudome, Kentaro Ushijima, Naoya Matsunaga, Satoru Koyanagi, Shigehiro Ohdo

**Affiliations:** 1 Department of Pharmaceutics, Faculty of Pharmaceutical Sciences, Kyushu University, Fukuoka, Japan; 2 Department of Clinical Pharmacokinetics, Faculty of Pharmaceutical Sciences, Kyushu University, Fukuoka, Japan; 3 Division of Pharmaceutics, Faculty of Pharmaceutical Sciences, Sanyo-Onoda City University, Sanyoonoda, Yamaguchi, Japan

**Keywords:** cysteine, cancer, cell cycle, translation, xCT inhibitor

## Abstract

Malignant cells exhibit a high demand for amino acids to sustain their abnormal proliferation. Particularly, the intracellular accumulation of cysteine is often observed in cancer cells. Previous studies have shown that deprivation of intracellular cysteine in cancer cells results in the accumulation of lipid peroxides in the plasma membrane and induction of ferroptotic cell death, indicating that cysteine plays a critical role in the suppression of ferroptosis. Herein, we found that the oncogenic accumulation of cysteine also contributes to cancer cell proliferation by promoting the cell cycle progression, which is independent of its suppressive effect on ferroptosis. The growth ability of four types of cancer cells, including murine hepatocarcinoma cells, but not of primary hepatocytes, were dependent on the exogenous supply of cysteine. Deprivation of intracellular cysteine in cancer cells induced cell cycle arrest at the G0/G1 phase, accompanied by a decrease in the expression of cyclin D1 and D2 proteins. The cysteine deprivation–induced decrease in D-type cyclin expression was associated with the upregulation of eukaryotic translation initiation factor 4E binding protein 1, which represses the translation of cyclin D1 and D2 proteins by binding to eukaryotic translation initiation factor 4E. Similar results were observed in hepatocarcinoma cells treated with erastin, an inhibitor of cystine/glutamate antiporter, xCT. These findings reveal an unappreciated role of cysteine in regulating the growth of malignant cancer cells and deepen our understanding of the cytotoxic effect of xCT inhibitor to prevent cancer cell proliferation.

Malignant cancers often alter their metabolism to maintain rapid growth and survive against various stresses such as oxidative damage, energy deprivation, and chemotherapeutic agents (1, 2). Specifically, cancer cells increase the demand for specific nutrients, depending on either the exogenous supply or upregulation of *de novo* biosynthesis (3). This nutrient requirement for the growth of cancer cells has been recognized as a potential target for the development of therapeutic drugs (4, 5).

Cysteine, a sulfur-containing amino acid, serves as a fundamental component in various biomolecules and plays a pivotal role in maintaining cellular functions, including protein synthesis, redox homeostasis, cell signaling, and detoxication (6). Intracellular accumulation of cysteine is often observed in various cancers, where it is used for glutathione biosynthesis to eliminate oxidative stress caused by rapid cell proliferation (7). The deprivation of intracellular cysteine in cancer cells leads to the accumulation of lipid peroxides in the cellular membrane, triggering ferroptosis, an iron-dependent form of cell death (8). Multiple studies have indicated that cysteine plays a crucial role in suppressing ferroptosis by regulating the glutathione peroxidase 4 expression and biosynthesis of glutathione or coenzyme A (9, 10). Ferroptosis has garnered significant attention in the scientific community as a novel mechanism of cell death in various diseases, including cancer, renal failure, Alzheimer’s disease, hepatic cirrhosis, and myocardial infarction (11, 12, 13). Thus, recent studies have focused on elucidating the role of cysteine as a regulator of ferroptosis (14, 15, 16).

Although many cancer cells enhance glutathione biosynthesis from cysteine, some cancer cells downregulate this reaction, despite an accumulation of intracellular cysteine (17, 18). These reports indicate other roles for cysteine in the regulation of cancer malignancy, but its pathological role in cancer cells is not fully understood. In this study, we investigated the pathological role of accumulated cysteine in four types of murine cancer cells, namely BNL 1ME A.7 R.1 hepatocarcinoma, 4T1 breast cancer, RenCa renal adenocarcinoma, and Colon-26 adenocarcinoma. Our findings reveal the unraveling role of cysteine in the cell cycle progression and the mechanism for enabling rapid proliferation of cancer cells.

## Results

### The exogenous cysteine supply dependency in cancer cell proliferation

Cancer cells increase the demand for various amino acids (2, 3, 19). We used four types of murine cancer cells (BNL 1ME A.7 R.1 hepatocarcinoma, 4T1 breast cancer, RenCa renal adenocarcinoma, and Colon-26 adenocarcinoma) and investigated the dependence of their proliferation on amino acids. Cells were incubated for 48 h in single amino acid–deficient media by removing each of the 15 amino acids from the culture media, and their growth was measured following medium replacement. Among the essential amino acids, deprivation of methionine and valine significantly suppressed the growth of all four types of cancer cells (Fig. S1). In addition, significant growth suppression of all the cancer cell types was observed when cells were incubated in the media deficient in glutamine, arginine, and cystine, although they are not essential amino acids.

**Figure 1:**
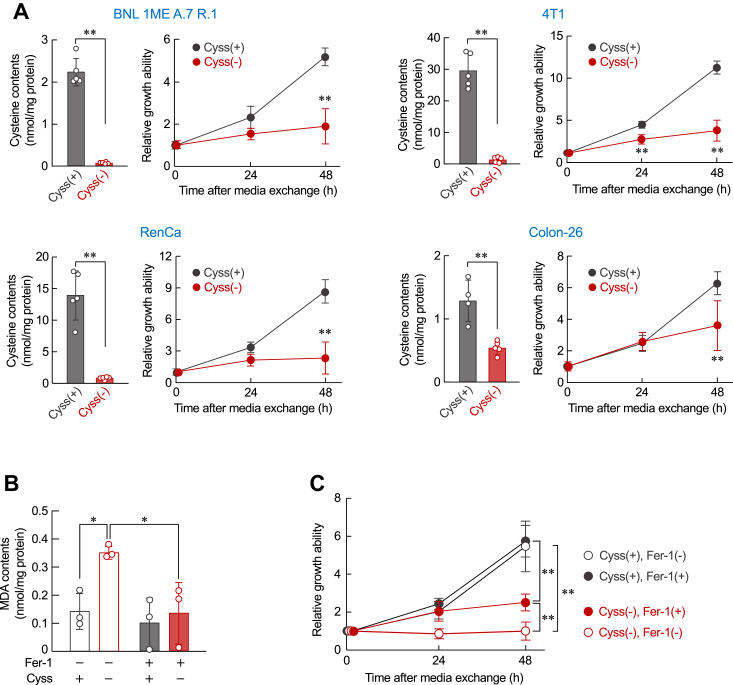
**The growth of cancer cells depends on the exogenous cysteine supply.***A*, cysteine-dependent growth of cancer cells. *Left columns* show intracellular cysteine levels at 16 h after incubation for four types of murine cancer cells in cystine-containing [Cyss (+)] or cystine-deficient [Cyss (−)] media. Intracellular cysteine contents were normalized by protein levels. Each value represents the mean with SD (n = 4–5). ∗∗*p* < 0.01, significant difference between the two groups (*t*_*8*_ = 14.924, *p* < 0.001 for BNL 1ME A.7 R.1 cells; *t*_*8*_ = 11.292, *p* < 0.001 for 4T1 cells; *t*_*8*_ = 7.518, *p* < 0.001 for RenCa cells; *t*_*7*_ = 4.879, *p* = 0.002 for Colon-26 cells; Student’s *t* test). *Right line graph* shows the growth ability of cells. The basal cell viability of the Cyss (+) media group (0 h) was set at 1.0. Each value represents the mean with SD (n = 6). ∗∗*p* < 0.01, significant difference from Cyss (+) groups at corresponding time points (*F*_2,30_ = 41.510, *p* < 0.001 for BNL 1ME A.7 R.1 cells; *F*_2,30_ = 105.449, *p* < 0.001 for 4T1 cells; *F*_2,30_ = 47.843, *p* < 0.001 for RenCa cells; *F*_2,30_ = 12.181, *p* < 0.001 for Colon-26 cells; two-way ANOVA with Tukey–Kramer’s post hoc test). *B*, intracellular contents of MDA in BNL 1ME A.7 R.1 cells after treatment with 10 μM ferrostatin-1 (Fer-1) in Cyss (+) or Cyss (−) media for 24 h. The intracellular contents of malondialdehyde (MDA) were normalized to protein levels. Each value represents the mean with SD. (n = 3). ∗*p* < 0.05, significant difference between the two groups (*F*_3,8_ = 6.345, *p* = 0.017; ANOVA with Tukey–Kramer’s post hoc test). *C*, the growth ability of BNL 1ME A.7 R.1 cells during treatment with 10 μM Fer-2 in Cyss (+) or Cyss (−) media. The basal cell viability of the Cyss (+) media group (0 h) was set at 1.0. Each value represents the mean with SD. (n = 4). ∗∗*p* < 0.01, significant difference between the two groups at corresponding time points (*F*_6__,__36_ = 57.951, *p* < 0.001; two-way ANOVA with Tukey–Kramer’s post hoc test).

The differences in cancer cell growth depending on the type of amino acid prompted us to investigate the underlying mechanism. Intracellular cysteine levels are maintained by two pathways, an exogenous supply in the oxidized form (*i.e.,* cystine) through the xCT transporter (cystine/glutamate antiporter) and an intracellular supply synthesized from methionine through the transsulfuration pathway (20). Since cancer cells undergo rapid cell proliferation and are exposed to high oxidative stress, they are thought to increase the demand for cysteine using glutathione biosynthesis (7, 21). Considering these points, we focused on the role of cysteine in the regulation of cancer cell proliferation. Deprivation of cystine in the culture medium significantly decreased the intracellular cysteine levels in all four types of cancer cells, accompanied by their growth suppression (Fig. 1*A*). Similar results have also observed in various human cancer cell lines in our previous study (22). The intracellular cysteine levels in primary hepatocytes prepared from BALB/c mice were decreased by approximately 50% following the deprivation of cystine in the culture medium, but the treatment had a negligible effect on their viability (Fig. S2*A*). Since BNL 1ME A.7 R.1 cells are generated by the treatment of BALB/c mice–derived hepatocytes with a chemical carcinogen (23), these results suggest that the oncogenic transformation of hepatocytes alters cysteine metabolism to sustain their rapid proliferation.

As cysteine is a rate limiting substrate for glutathione synthesis, oncogenic accumulation of cysteine is thought to prevent ferroptotic cell death following oxidative stress (24, 25). The deprivation of cystine in the cultured media of BNL 1ME A.7 R.1 cells increased the levels of malondialdehyde (MDA), a reliable marker of lipid peroxidation associated with ferroptosis. Although the accumulation of MDA was significantly suppressed by treating BNL 1ME A.7 R.1 cells with ferroptosis inhibitor ferrostatin-1 (Fer-1) (Fig. 1*B*), a supplement of Fer-1 in cystine-deficient media failed to restore their growth ability to the level observed when cultured in cystine-containing media (Fig. 1*C*). These results indicate that accumulated cysteine in oncogenic transformed hepatocytes not only prevents ferroptosis but also sustains the cell proliferation.

### Cysteine deprivation induces G0/G1 phase arrest of the cell cycle by decreasing the expression of D-type cyclins

**Figure 2:**
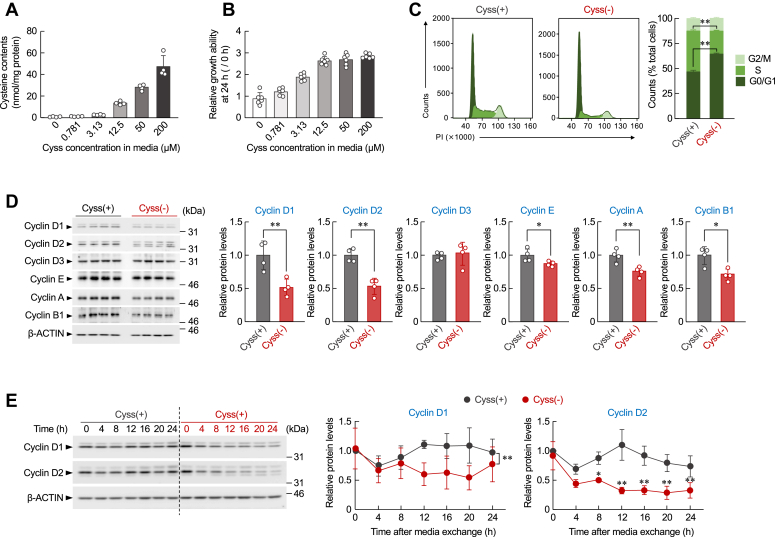
**Cysteine-dependent cell cycle progression of BNL 1ME A.7 R.1 cells.***A* and *B*, the intracellular contents of cysteine (*A*) and growth ability (*B*) of BNL 1ME A.7 R.1 cells increase in an extracellular cysteine concentration-dependent manner. Cells were incubated in conditional media containing the indicated concentrations of cystine for 24 h. Intracellular contents of cysteine were normalized to protein levels. Each value represents the mean with SD (n = 4–6). *C*, representative images (*left*) and quantification (*right*) of the cell cycle distribution in BNL 1ME A.7 R.1 cells incubated in cystine-containing [Cyss (+)] or cystine-deficient [Cyss (−)] media for 24 h. Each value represents the mean with SD (n = 4). ∗∗*p* < 0.01, significant difference between the two groups (*t*_6_ = −32.705, *p* < 0.001 for G0/G1 phase; *t*_6_ = 56.454, *p* < 0.001 for S phase; Student’s *t* test). *D*, the protein levels of cyclins in BNL 1ME A.7 R.1 cells 24 h after incubation in Cyss (+) or Cyss (−) media. The protein levels were normalized to those of β-actin. Each value represents the mean with SD (n = 4). ∗∗*p* < 0.01; ∗*p* < 0.05, significant difference between the two groups (*t*_6_ = 3.883, *p* = 0.008 for cyclin D1; *t*_6_ = 5.983, *p* = 0.001 for cyclin D2; *t*_6_ = 2.478, *p* = 0.048 for cyclin E; *t*_6_ = 4.090, *p* = 0.006 for cyclin A; *t*_6_ = 3.580, *p* = 0.012 for cyclin B1; Student’s *t* test). *E*, temporal expression profiles of cyclin D1 and cyclin D2 protein levels in BNL 1ME A.7 R.1 cells during the incubation in Cyss (+) or Cyss (−) media. The protein levels were normalized to those of β-actin. Each value represents the mean with SD (n = 4). ∗∗*p* < 0.01, ∗*p* < 0.05, significant difference between the two groups (*F*_1,42_ = 18.585, *p* < 0.001 for cyclin D1; *F*_6,42_ = 5.316, *p* < 0.001 for cyclin D2; two-way ANOVA with Tukey–Kramer’s post hoc test).

When BNL 1ME A.7 R.1 cells were incubated in media containing various concentrations of cystine, the growth of cells was increased in an intracellular cysteine level–dependent manner (Fig. 2, *A* and *B*). To investigate the underlying mechanism of cysteine deprivation–induced suppression of cancer cell growth, we analyzed the cell cycle distribution of BNL 1ME A.7 R.1 cells incubated in cystine-containing or cystine-deficient media for 24 h. The deprivation of cysteine induced G0/G1 phase arrest of the cell cycle (Fig. 2*C*). The treatment also decreased the protein levels of four types of cyclins (A, B, D, and E) in hepatocarcinoma cells (Fig. 2*D*). Among them, the expression levels of cyclin D1 (*Ccnd1*) and *Ccnd*2 proteins were more markedly decreased compared with other types of cyclins. The protein levels of these D-type cyclins were decreased depending on cystine concentrations in cultured media (Fig. S3). Similar decreases in the protein levels of *Ccnd1* and *Ccnd2* were also observed in 4T1, RenCa, and Colon-26 cells when they were incubated in cystine-deficient media (Fig. S4). In contrast, neither the *Ccnd1* nor *Ccnd*2 protein levels in primary hepatocytes were altered by cysteine deprivation (Fig. S2*B*). Although transduction of BNL 1ME A.7 R.1 cells with lentivirus expressing *Ccnd1* and *Ccnd1*2 increased their protein levels, cysteine deprivation–induced cell cycle arrest was rescued by enhanced expression of cyclin D (Fig. S5). Since D-type cyclins are key regulators in the control of cell cycle progression from the G0/G1 to S phase (26), the present results indicate that oncogenic accumulation of cysteine contributes to the rapid proliferation of hepatocarcinoma cells by promoting the expression of *Ccnd1* and *Ccnd2* proteins.

### Cysteine deprivation induces nuclear accumulation of mRNAs for *Ccnd1* and *Ccnd2*

**Figure 3:**
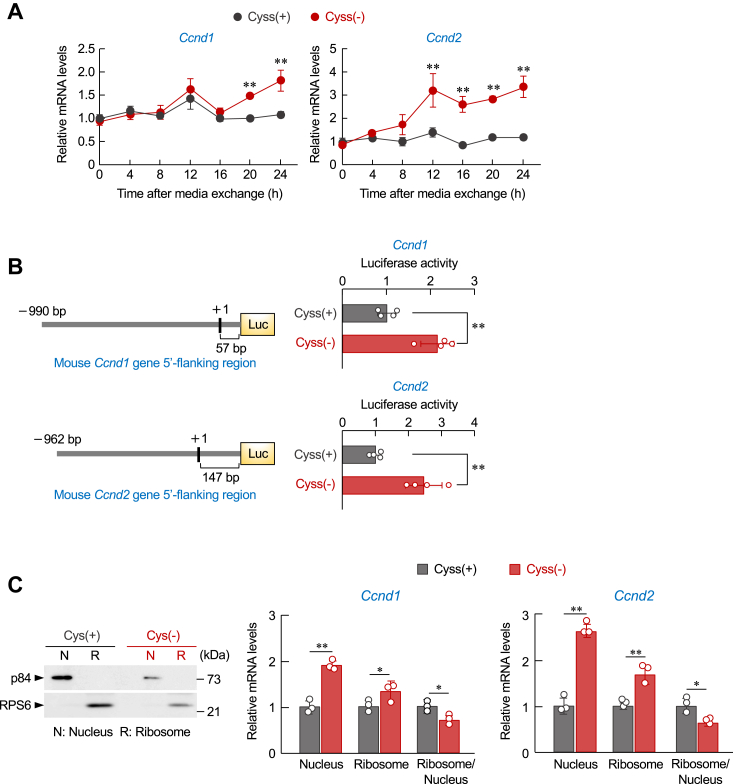
**Nuclear accumulation of *Ccnd1* and *Ccnd2* mRNAs by cysteine deprivation.***A*, temporal expression profiles of *Ccnd1* and *Ccnd2* mRNAs in BNL 1ME A.7 R.1 cells during the incubation in cystine-containing [Cyss (+)] or cystine-deficient [Cyss (−)] media. The mRNA levels were normalized to those of *β-actin*. Each value represents the mean with SD (n = 4). ∗∗*p* < 0.01, significant difference from the Cyss (+) group at corresponding time points (*F*_6,42_ = 10.290, *p* < 0.001 for *Ccnd1*, *F*_6,42_ = 19.431, *p* < 0.001 for *Ccnd2*; two-way ANOVA with Tukey–Kramer’s post hoc test). *B*, Luciferase activity of *Ccnd1* gene promotor-driven reporter and *Ccnd2* gene promotor-driven reporter in BNL 1ME A.7 R.1 cells 24 h after incubation in Cyss (+) or Cyss (−) media. The luciferase activities were normalized to those driven from firefly luciferase reporter (pRL-TK) vectors. Each value represents the mean with SD (n = 4). ∗∗*p* < 0.01, significant difference between the two groups (*t*_6_ = 5.343, *p* = 0.002 for *Ccnd1*; *t*_6_ = 0.002, *p* = 5.028 for *Ccnd2*; Student’s *t* test). *C*, BNL 1ME A.7 R.1 cells were incubated in Cyss (+) or Cyss (−) media for 24 h, and then the nuclei and the ribosomes were extracted. *Left panel* shows Western blotting images of p84 (nuclear marker) and RPS6 (ribosome marker) prepared from BNL 1ME A.7 R.1 cells. *Right panel* shows mRNA levels for *Ccnd1* and *Ccnd2* in the nuclei and ribosomes of BNL 1ME A.7 R.1 cells. The mRNA levels were normalized to those of *18s*. Each value represents the mean with SD (n = 3). ∗∗*p* < 0.01, ∗*p* < 0.05; significant difference between the two groups (*t*_4_ = −13.828, *p* < 0.001 for *Ccnd1* nuclei; *t*_4_ = −3.281, *p* = 0.031 for *Ccnd1* ribosome; *t*_4_ = 4.209, *p* = 0.014 for *Ccnd1* ribosome/nuclei; *t*_4_ = −12.476, *p* < 0.001 for *Ccnd2* nucleus; *t*_4_ = −5.377, *p* = 0.006 for *Ccnd2* ribosome; *t*_4_ = 3.626, *p* = 0.022 for *Ccnd2* ribosome/nuclei; Student’s *t* test). *Ccnd*, cyclin D.

Protein levels are regulated by transcription, translation, and degradation processes. The expression levels of *Ccnd1* and *Ccnd2* proteins gradually decreased after replacement with cystine-deficient media (Fig. 2*E*), whereas their mRNA levels were increased by cysteine deprivation (Fig. 3*A*). To further investigate whether cysteine deprivation increases transcriptional activity of the *Ccnd1* and *Ccnd2* genes, we constructed luciferase reporter vectors containing the mouse *Ccnd1* or *Ccnd2* gene upstream regions. BNL 1ME A.7 R.1 cells were transfected with these reporter vectors and then incubated in cystine-containing or cystine-deficient media (Fig. 3*B*). The luciferase activities driven by each *Ccnd1* and *Ccnd2* gene upstream region were increased by cysteine deprivation. These results indicated that both *Ccnd1* and *Ccnd2* mRNAs are accumulated in cancer cells under the cysteine depletion condition. The increasing amount of mRNAs for *Ccnd1* and *Ccnd2* was detected in both the nuclear and ribosomal fractions when BNL 1ME A.7 R.1 cells were incubated in cystine-deficient media, but the ratio of mRNA content of *Ccnd1* and *Ccnd2* in ribosomes to nuclear content was significantly decreased by cysteine deprivation (Fig. 3*C*). Overall, the data suggested that the depletion of cysteine suppresses the translation of *Ccnd1* and *Ccnd2* proteins by preventing the translocation of their mRNAs from the nucleus to the cytoplasm.

### Cysteine deprivation suppresses the translation of *Ccnd1* and *Ccnd2* by enhancing the expression of 4E-BP1

**Figure 4:**
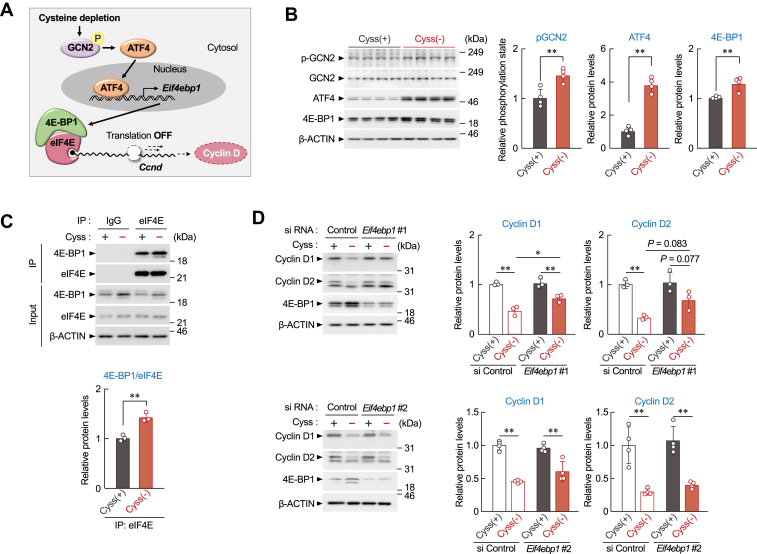
**4E-BP1 is involved in cysteine deprivation-induced repression of D-type cyclin translation.***A*, schematic diagram of hypothesis for cysteine-mediated regulation for the translation of D-type cyclins. *B*, the protein levels of pGCN2, ATF4, and 4E-BP1 in BNL 1ME A.7 R.1 cells 16 h after incubation in cystine-containing [Cyss (+)] or cystine-deficient [Cyss (−)] media. The protein levels were normalized to those of total GCN2 (for pGCN2) or β-actin (for ATF4 and 4E-BP1). Each value represents the mean with SD (n = 4). ∗∗*p* < 0.01, significant difference between the two groups (*t*_6_ = −3.958, *p* = 0.008 for pGCN2; *t*_6_ = −10.744, *p* < 0.001 for ATF4; *t*_6_ = −4.046, *p* = 0.007 for 4E-BP1; Student’s *t* test). *C*, the protein levels of 4E-BP1 bound to eIF4E in BNL 1ME A.7 R.1 cells 16 h after incubation in Cyss (+) or Cyss (−) media. Cell extracts were immunoprecipitated with anti-eIF4E antibodies. The protein levels were normalized to those of eIF4E. Each value represents the mean with SD (n = 3). ∗∗*p* < 0.01, significant difference between the two groups (*t*_6_ = −6.678, *p* = 0.003; Student’s *t* test). *D*, downregulation of 4E-BP1 restores cysteine deprivation–induced repression cyclin D1 and D2 protein levels in BNL 1ME A.7 R.1 cells. Cells were transfected with scrambled siRNA (control) or two types of siRNA against *Eif4ebp1* gene encoding 4E-BP1 (*Eif4ebp1*#1 and *Eif4ebp1*#2) and then incubated in Cyss (+) or Cyss (−) media for 16 h. The protein levels were normalized to those of β-actin. Each value represents the mean with SD (n = 3–4). For *upper panels*, ∗∗*p* < 0.01, ∗*p* < 0.05, significant difference between the two groups (*F*_3,8_ = 32.627, *p* < 0.001 for cyclin D1, *F*_3,8_ = 14.316, *p* < 0.001 for cyclin D2; ANOVA with Tukey–Kramer’s post hoc test). For *lower panels*, ∗∗*p* < 0.01, significant difference between the two groups (*F*_3,12_ = 35.369, *p* < 0.001 for cyclin D1; *F*_3,12_ = 20.061, *p* < 0.001 for cyclin D2; ANOVA with Tukey–Kramer’s post hoc test). 4E-BP, 4E-binding protein; ATF4, activating transcription factor-4; eIF4, eukaryotic translation initiation factor 4; GCN2, general control nonderepressible 2.

General control nonderepressible 2 (GCN2) kinase, an amino acid deprivation sensor, regulates the expression and activity of genes involved in stress response by inducing the expression of activating transcription factor-4 (ATF4) (27). ATF4 also functions as a transcriptional regulator of eukaryotic translation initiation factor 4E (eIF4E)-binding protein 1 (4E-BP1) (28, 29). Although eIF4E binds to the 5′ cap structure of mRNA and promotes the initial step of translation of most proteins in eukaryotes (30), the synthesis of D-type cyclins is highly dependent on this translational regulator (31, 32). Nonphosphorylated 4E-BP1 binds to eIF4E, preventing its assembly into the eIF4G complex and inhibiting cap-dependent translation (28, 29). Based on these facts, we hypothesized that cysteine depletion enhances 4E-BP1 expression by activating the GCN2/ATF4 signaling pathway and suppresses *Ccnd1* and *Ccnd2* protein expression by promoting the 4E-BP1/eIF4E interaction (Fig. 4*A*). The phosphorylation state of GCN2 and the protein levels of ATF4 and 4E-BP1 increased in BNL 1ME A.7 R.1 cells under the cysteine depletion condition (Fig. 4*B*). The results of coimmunoprecipitation also showed that the amount of eIF4E bound to 4E-BP1 was significantly increased by cysteine deprivation (Fig. 4*C*). Furthermore, downregulation of 4E-BP1 in BNL 1ME A.7 R.1 cells by siRNA restored the cysteine depletion–induced suppression of *Ccnd1* and *Ccnd2* protein expression (Fig. 4*D*). On the other hands, enhanced expression of GCN2 suppressed the growth of BNL 1ME A.7 R.1 cells (Fig. S6*A*) accompanied by suppressing D-type cyclin expression (Fig. S6*B*). These results support our hypothesis that cysteine depletion decreases the *Ccnd1* and *Ccnd2* protein levels by repressing their translation.

### The suppressive effect of erastin on cancer cell growth involves G0/G1 cell cycle arrest and downregulation of *Ccnd1* and *Ccnd2* proteins

**Figure 5:**
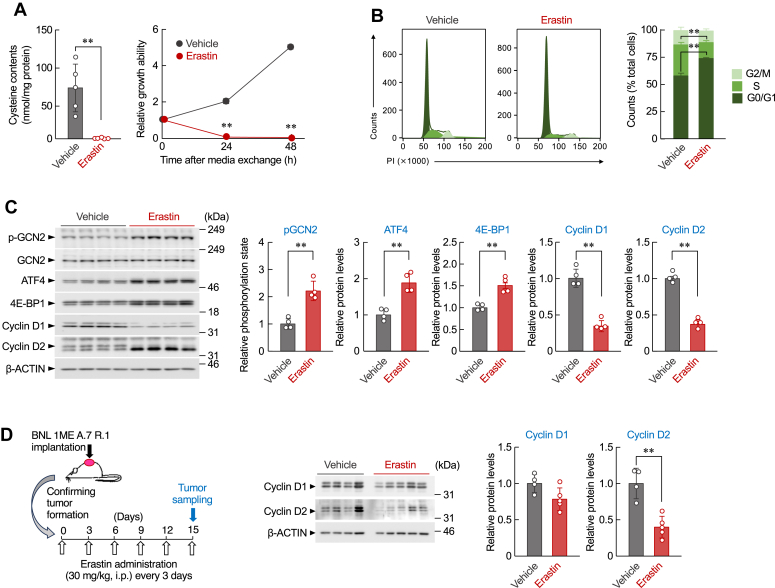
**Pharmacological inhibition of extracellular cystine uptake by erastin prevents the growth of BNL 1ME A.7 R.1 by suppressing cyclin D1 and D2 protein expression.***A*, intracellular cysteine contents (*left*) and growth ability (*right*) of BNL 1ME A.7 R.1 cells after treatment with 5 μM erastin. *Left column* shows intracellular cysteine contents in cells 16 h after erastin treatment. Each value represents the mean with SD (n = 4). ∗∗*p* < 0.01, significant difference between the two groups (*t*_8_ = 4.690, *p* = 0.002; Student’s *t* test). *Right line graph* shows the growth ability of cells. The basal cell viability of the vehicle group (0 h) was set to 1.0. Each value represents the mean with SD (n = 4). ∗∗*p* < 0.01, significant difference from the vehicle-treated group at corresponding time points (*F*_2,18_ = 1853.981, *p* < 0.001; two-way ANOVA with Tukey–Kramer’s *post hoc* test). *B*, representative images (*left*) and quantification (*right*) of the cell cycle distribution in BNL 1ME A.7R.1 cells incubated in 5 μM erastin for 24 h. Each value represents the mean with SD (n = 4). ∗∗*p* < 0.01, significant difference between the two groups (*t*_6_ = −14.240, *p* < 0.001 for G0/G1 phase; *t*_6_ = 11.748, *p* < 0.001 for S phase; Student’s *t* test). *C*, the protein levels of pGCN2, ATF4, 4E-BP1, cyclin D1, and cyclin D2 in BNL 1ME A.7 R.1 cells after treatment with vehicle or 5 μM erastin for 24 h. The protein levels were normalized to those of total GCN2 (for pGCN2) or β-actin (for ATF4, 4E-BP1, cyclin D1, and cyclin D2). Each value represents the mean with SD (n = 4). ∗∗*p* < 0.01, significant difference between the two groups (*t*_6_ = −5.963, *p* = 0.001 for pGCN2; *t*_6_ = −5.686, *p* = 0.001 for ATF4; *t*_6_ = −5.303, *p* = 0.002 for 4E-BP1 *t*_6_ = 9.226, *p* < 0.001 for cyclin D1; *t*_6_ = 13.858, *p* < 0.001 for cyclin D2; Student’s *t* test). *D*, the protein levels of cyclin D1 and cyclin D2 in BNL 1ME A.7 R.1–fromed tumors in mice after intraperitoneally (*i.p.*) administration of vehicle or erastin (30 mg/kg). *Left panel* indicates the experimental protocol. *Right panel* show the protein levels of D-type cyclins. Their protein levels were normalized to those of β-actin. Each value represents the mean with SD (n = 4–5). ∗∗*p* < 0.01, significant difference between the two groups (*t*_7_ = −5.027, *p* = 0.002; Student’s *t* test). 4E-BP, 4E-binding protein; ATF4, activating transcription factor-4; GCN2, general control nonderepressible 2.

Erastin is a classic ferroptosis inducer that suppresses xCT, subsequently inhibits cellular cystine uptake, and suppresses the growth of several types of cancer cells (8). In the final set of experiments, we investigated whether the cell cycle arrest and downregulation of *Ccnd1* and *Ccnd2* proteins were also involved in the suppressive effect of erastin on the growth of BNL 1ME A.7 R.1 cells. BNL 1ME A.7 R.1 cells treated with 5 μM erastin exhibited decreased intracellular cysteine levels, accompanied by inhibited cell growth (Fig. 5*A*). A similar phenomenon was observed when cells were incubated in cystine-deficient media, and erastin treatment increased the distribution of cells in the G0/G1 phase of the cell cycle (Fig. 5*B*). Furthermore, significant decreases in the *Ccnd1* and *Ccnd2* protein levels were detected in erastin-treated hepatocarcinoma cells, while the phosphorylation state of GCN2 and the expressions of ATF4 and 4E-BP1 were increased (Fig. 5*C*). Erastin also decreased the cyclin D1 and D2 protein levels in 4T1, RenCa, and Colon-26 cells (Fig. S7). In addition, suppression of D-type cyclin expressions was observed by erastin administration (30 mg/kg body weight, i.p) in BNL 1ME A.7 R.1–formed tumors in mice (Fig. 5*D*). These results indicate that pharmacological inhibition of the exogenous cysteine supply by erastin prevents cancer cell proliferation. The suppressive effect involves G0/G1 cell cycle arrest and downregulation of the *Ccnd1* and *Ccnd2* proteins.

## Discussion

**Figure 6:**
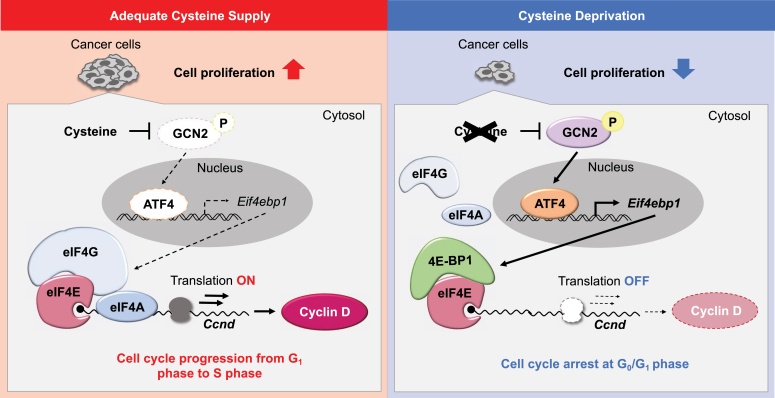
**Oncogenic accumulation of cysteine promotes the cell cycle progression by regulating the translation of cyclin D1 and D2 proteins.** Cysteine deprivation induces cell cycle arrest at the G0/G1 phase in cancer cells, resulting from the suppressed translation of cyclin D1 and D2 proteins. Cysteine deprivation–induced repression of D-type cyclin expression is associated with the prevention of eIF4E-mediated translation machinery by upregulating 4E-BP1. 4E-BP, 4E-binding protein; eIF4, eukaryotic translation initiation factor 4.

Due to their abnormal growth and rapid proliferation, cancer cells are exposed to high oxidative stress and exhibit an increased demand for cysteine (21). Accumulation of intracellular cysteine in cancer cells is thought to be used for antioxidant synthesis and the avoidance of ferroptosis (33). In this study, we found that intracellular accumulated cysteine contributes to cancer cell proliferation by promoting cell cycle progression. In addition to the induction of ferroptosis, the reduction in intracellular cysteine levels also arrested the cell cycle of hepatocarcinoma cells in the G0/G1 phase, accompanied by decreases in the *Ccnd1* and *Ccnd2* protein levels. The decrease in the expression of D-type cyclins was due to the repression of their translation associated with the entrapment of eIF4E by 4E-BP1 (Fig. 6).

The cysteine supply in cancer cells often depends on exogenous uptake through cystine transporter xCT (34, 35). Herein, the intracellular cysteine levels of all four types of murine cancer cells and their proliferation were significantly decreased when cells were incubated in cystine-deficient media, but the treatment had a negligible effect on the viability of mouse primary hepatocytes whose growth ability was modest than oncogenic-transformed cells. This is probably due to the difference in cysteine metabolism between malignant cancers and normal cells. The expressions of xCT were higher in cancer cells than in normal cells (Fig. S8*A*), whereas the expressions of enzymes responsible for *de novo* cysteine synthesis, cystathionine-β-synthase (CBS), and cystathionine-γ-lyase (CTH), were lower in cancer cells than in healthy hepatocytes (Fig. S8*B*). We recently reported that epigenetic repression of *de novo* cysteine synthases in hepatocarcinoma cells upregulates the expression of xCT and subsequent intracellular cysteine accumulation (36). In the report, we showed that inhibition of *de novo* cysteine synthesis in primary hepatocytes by treatment with CTH inhibitor propargylglycine increases the expression of *Slc7a11* mRNA and xCT protein *via* accumulation of NF-E2-related factor 2. Therefore, disruption of intracellular cystine synthesis pathway appears to indirectly confer abnormal proliferative ability on oncogenic transformed cells. On the other hand, deprivation of exogenous cysteine supply had a negligible effect on the expression of enzymes responsible for *de novo* cysteine synthesis in primary hepatocytes (Fig. S8*C*), indicating that oncogenic disruption of endogenous cysteine synthesis is an irreversible, and this disruption does not appear to be an event caused by alterations in extracellular cysteine levels. Taken together, the oncogenic alteration of cysteine metabolism highlights the exogenous cystine supply pathway as a viable therapeutic target in cancer.

Intracellular cysteine depletion induces ferroptosis in various cancers such as B cell lymphoma, renal cancer, and pancreatic cancer (9, 10, 24). Cysteine deprivation causes a decrease in the levels of intracellular antioxidants, including glutathione, and an accumulation of lipid peroxides in the plasma membrane. Fer-1, a lipid radical scavenger, is often used as an inhibitor of ferroptosis (37). Although Fer-1 treatment significantly suppressed cysteine deprivation-induced ferroptosis in BNL 1ME A.7 R.1 cells, it did not restore their growth activity. The treatment also causes cell cycle arrest at the G0/G1 phase and a decrease in *Ccnd1* and *Ccnd2* protein expression. Notably, D-type cyclins are recognized for their involvement in human oncogenesis (38). The expression of *Ccnd3* was not altered by cysteine deprivation. Since the expression level of each D-type cyclin was different depending on oncogenesis (39, 40, 41), *Ccnd3* may not be abundant in our examined cancer cells (42). Excessive expression of D-type cyclins is a trigger for abnormal cell proliferation because D-type cyclins promote the progression of the cell cycle from the G0/G1 to S phase by inducing cyclin E and A expression (43, 44). Therefore, cysteine deprivation–induced repression of cyclin E, A, and B may be attributed to the decrease in *Ccnd1* and *Ccnd2* protein levels.

Despite decreasing the protein levels of *Ccnd1* and *Ccnd2*, cystine-deficient media increased the mRNA levels and transcription activity of those D-type cyclins. The deprivation of cysteine increased the mRNA levels of transcription factor *YB-1* in BNL 1ME A.7 R.1 cells (Fig. S9*A*). Increased levels of *YB-1* have been observed in several types of cancers and contribute to cell growth (45). *YB-1* binds to Y-box in the upstream region of the *Ccnd1* gene and promotes its transcription (46). Several consensus sequences of Y-box (CAAT) are located in the mouse *Ccnd1* and *Ccnd2* genes (Fig. S9*B*). Although it remains to be clarified why cysteine deprivation increases *YB-1* expression, enhanced expression of this transcription factor seemed to increase the transcription of *Ccnd1* and *Ccnd2* under cysteine depletion conditions.

The process of mRNA translation comprises initiation, extension, and termination, and the initiation step is strictly regulated (47). eIF4E, which regulates the initiation of mRNA translation, binds with the 5′-cap structure and recruits scaffolding protein eIF4G and RNA helicase eIF4A. These triple complexes formed by eIF4E, eIF4G, and eIF4A induce mRNA translation (30). 4E-BP1 has higher affinity for eIF4E than eIF4G. The inhibition of this protein regulates cap-dependent mRNA translation by preventing eIF4E–eIF4G–eIF4A complexes from forming (29, 32). Although the 5′ m(7)G cap moiety on all mRNAs is sufficient for their functional interaction with eIF4E, several mRNAs, including *Ccnd1*, have long and complex secondary structures in the 5′-UTR, which is preferentially translated in an eIF4E-dependent manner (48). Furthermore, eIF4E promotes the nuclear export of *Ccnd1* mRNAs through an element in the 3′-UTR (49). Incubation of BNL 1ME A.7 R.1 cells in cystine-deficient media increased the expression of 4E-BP1 and its binding amount to eIF4E, indicating that cysteine deprivation prevents eIF4E functions. Therefore, enhanced expression of 4E-BP1 in BNL 1ME A.7 R.1 cells after incubation in cystine-deficient media may not only prevent the translation of *Ccnd1* and *Ccnd2* proteins but also cause their mRNA accumulation in nuclei.

However, downregulation of 4E-BP1 partially restored the expression levels for *Ccnd1* and *Ccnd2* proteins, suggesting that other factors also participate in the regulation of D-type cyclin expression under cysteine depletion conditions. The helicase activity of eIF4A remains low under normal conditions, but its activity is stimulated by the binding of eIF4B (50). The activity of eIF4B is regulated depending on ribosomal protein S6 kinase–mediated phosphorylation. The S6 kinase activity was shown to decrease in human colorectal cancer cells cultured with cystine-deficient media (27), suggesting that eIF4B is also involved in the cysteine-dependent translational regulation of D-type cyclins. The protein expression of D-type cyclins, c-MYC, ornithine decarboxylase, and vascular endothelial growth factor are also highly dependent on eIF4E-mediated translational machinery (31, 32, 51). These proteins participate in the regulation of cancer cell survival and proliferation. We observed that c-MYC expression was decreased in BNL 1ME A.7 R.1 cells when cells were incubated in cystine-deficient media (Fig. S10). Similar to D-type cyclins, translation of the cell survival and proliferation regulators may also be controlled by the GCN2/ATF4 signaling pathway.

Treatment of BNL 1ME A.7 R.1 cells with erastin suppressed the growth and expressions of D-type cyclins, as observed when cells were cultured in cystine-deficient media. In addition to the inhibition of cystine uptake, erastin also activates p53 and promotes the opening of voltage-dependent anion channels in mitochondria (52, 53). These multiple actions of erastin are thought to be involved in its cytotoxic effect. Erastin is a classic ferroptosis inducer. Treatment with erastin also increased the expression of ferroptosis marker, *Chac1* and *Ptgs2*, in BNL 1ME A.7 R.1 cells, suggesting that the xCT inhibitor exerts its cytotoxic effect on hepatocarcinoma cells by inducing cell cycle arrest and ferroptosis (Fig. S11*A*). Ferroptosis inhibitor Fer-1 attenuated the cytotoxic effect of erastin on BNL 1ME A.7 R.1 cells, but did not restore their growth completely (Fig. S11*B*). In addition, Fer-1 treatment also failed to restore erastin-induced cell cycle arrest (Fig. S11*C*), although neither erastin nor Fer-1 affected phosphorylation state of histone H2A.X (γ-H2AX), an index of DNA damage (Fig. S11*D*). These results supported our hypothesis that cancer cells increase the demand for cysteine to promote the cell cycle, as well as protect against ferroptotic cell death.

In normal healthy cells, cysteine is produced through the *de novo* synthesis pathway. However, the present findings show that maintaining cysteine levels in cancer cells largely depends on the cystine supply from the extracellular environment, which is consistent with previous reports. Although erastin effectively inhibits cystine uptake by xCT (54), its low solubility and instability in the body hinder its clinical application as an anticancer agent. To address these issues, a structure-modified compound called imidazole ketone erastin has been developed by adding imidazole and ketone groups (55). This erastin analog exhibits antitumor effects in B-cell lymphoma and renal cancer mouse models (56). In addition, sulfasalazine, which is used for treating ulcerative colitis, effectively inhibits cystine uptake by xCT and shows antitumor effects in cholangiocarcinoma and glioma mouse models (57, 58). As another approach, cyst(e)inase is an engineered synthetic enzyme that degrades both cystine and cysteine and can effectively deplete circulating cystine and cysteine in mice and humans with no obvious side effects (59). This degradation enzyme also exhibits antitumor effects on prostate cancer and pancreatic cancers (9, 59). These studies further suggest that the exogenous cysteine supply pathway is a viable therapeutic target in cancer treatment. In the present study, we highlighted the involvement of extracellular cysteine in regulating the cell cycle of cancer cells and demonstrated its contribution to the anticancer effects of erastin. The present results suggest the potential novel therapeutic strategies involving a combination of anticancer agents targeting the cell cycle and inhibitors targeting the cysteine supply pathway.

## Experimental procedures

### Cell culture and treatment

Murine hepatic cancer cell line BNL 1ME A.7 R.1 (RRID: CVCL_6371) and murine breast cancer cell line 4T1 (RRID: CVCL_0125) were obtained from American Type Culture Collection. Murine renal cancer cell line RenCa (RRID: CVCL_2174) was obtained from Cell Line Service. Murine colorectal cancer cell line Colon-26 (RRID: CVCL_0240) was obtained from the Cell Resource Center for Biomedical Research, Tohoku University. BNL 1ME A.7 R.1 cells were maintained in standard Dulbecco’s modified eagle medium (DMEM; Sigma-Aldrich). 4T1 cells, RenCa cells, and Colon-26 cells were maintained in standard RPMI (Thermo Fisher Scientific). The media was supplemented with 5% fetal bovine serum (FBS; Biowest) and 0.25% penicillin-streptomycin (Thermo Fisher Scientific). Cells were maintained at 37 °C in a humidified 5% CO_2_ atmosphere. We confirmed the absence of microbes in these cell lines using a MycoBlue *Mycoplasma* Detector (Nanjing Vazyme Biotech).

Cystine-containing and cystine-deficient media were prepared from Gibco DMEM with high glucose, but without glutamine, methionine, or cystine (#21013-024; Thermo Fisher Scientific) supplemented with 1 mM L-sodium pyruvate (Nacalai Tesque), 5% FBS, and 0.25% penicillin-streptomycin. L-methionine (Fujifilm Wako Pure Chemical Co) and L-glutamine (Tokyo Chemical Industry) were added to the media at final concentrations of 200 μM and 4 mM, respectively. To prepare cysteine-containing media, L-cystine dihydrochloride (Fujifilm Wako Pure Chemical) was added at a final concentration of 200 μM. Cells were seeded in culture plates and cultured in standard medium (DMEM containing 5% FBS and 0.25% penicillin-streptomycin) for 24 h. After the confirmation of cell attachment, the standard media were replaced with cystine-containing media or cystine-deficient media. Cells were also treated with erastin (SML2794; Sigma-Aldrich) or Fer-1 (F1302; Tokyo Chemical Industry). As a vehicle control, cells were treated with 0.05% (v/v) dimethyl sulfoxide (DMSO).

### Isolation and primary cell culture of hepatocytes

Hepatocytes were isolated from male BALB/c mice using a collagenase perfusion method (60, 61). Cells were plated on 6-well or 24-well plates at a density of 4 × 10^6^ or 8 × 10^5^ cells per well in Williams’ medium (Sigma-Aldrich) containing 5% FBS, 0.1 μM insulin (Fujifilm Wako Pure Chemical), 0.1 μM dexamethasone (Fujifilm Wako Pure Chemical), and 1% penicillin/streptomycin and incubated in 37 °C in a humidified 5% CO_2_ atmosphere. After the confirmation of cell attachment, cells were used for each experiment.

### Measurement of the intracellular cysteine concentrations

Cysteine in cultured cells was extracted using the Bligh–Dyer method. Cells were washed with saline and homogenized with 300 μl methanol containing 5 μl of internal standard (10 μg/ml L-[1-^13^C] valine). The homogenates were centrifuged for 10 min at 12,000*g* and 4 °C. Then, 100 μl of chloroform and 200 μl of supernatants were mixed with 5% (w/v) 5-sulfosalicylic acid. After 10 min of incubation at room temperature, samples were mixed with 100 μl of chloroform and 100 μl of water. The samples were centrifuged for 10 min at 1500*g* and 4 °C. Next, the upper phages were dried out and then dissolved into 50 μl of the mobile phase. A liquid chromatography tandem mass spectrometer (Waters) was used for the quantification of cysteine. Lipid chromatography was performed using an Intrada amino acid column (3 μm, 50 mm × 3 mm; Imtakt Co) under a gradient elution program. The mobile phase consisted of solvent A (acetonitrile – tetrahydrofuran – 25 mM ammonium formate – formic acid = 9:75:16:0.3, v/v/v/v) and solvent B (acetonitrile – 100 mM ammonium formate = 1:4, v/v). The following gradient program was applied: 2.5 min isocratic at 100% buffer A, 4 min gradient to 17% buffer B, 3.5 min isocratic at 100% buffer B, and 5 min isocratic at 100% buffer A with a flow rate of 0.6 ml/min. The mass spectrometer was operated in the multiple reaction monitoring mode using positive electrospray ionization. The multiple reaction monitoring was set at 122-76.1 and 119-72 *m/z* for cysteine and L-[1-^13^C] valine, respectively. The levels of metabolites were normalized to protein concentrations measured using a Pierce Bicinconic Acid Assay Kit (Thermo Fisher Scientific).

### Cell viability assay

Cells were seeded at a density of 1000 cells per well in 96-well plates. After incubation for 24 h, the media were exchanged with cystine-containing media or cystine-deficient media, and then further incubated in the presence or absence of 10 μM Fer-1 or 5 μM erastin. The compounds were dissolved in 0.05% (v/v) DMSO. Cell viability was assessed at the indicated time points using the CellTiter-Glo Luminescent Cell Viability Assay System (Promega) following the manufacturer’s instructions.

### Measurement of the intracellular MDA levels

After treatment of BNL 1ME A.7 R.1 cells with 10 μM Fer-1 or vehicle (0.05% DMSO) for 24 h, intracellular MDA levels were measured using an MDA Assay Kit (Dojindo) following the manufacturer’s instructions. Fluorescence was measured using an EnSpire Multimode Plate Reader (Ex: 540 nm, Em: 590 nm). The levels of MDA were normalized to protein concentrations measured using a Pierce Bicinconic Acid Assay Kit.

### Cell cycle analysis

At the time point of 24 h after media exchange, cells were treated with trypsin, washed with PBS, and fixed in 70% ethanol for 1 h at 4 °C. The fixed cells were washed with cold Hanks balanced salt solution containing 2% FBS, and then incubated in 1 mg/ml DNAase-free RNase for 1 h at 37 °C. The samples were washed again, and then incubated with 50 μg/ml propidium iodide at 4 °C for 2 h in the dark. Flow cytometric analysis was performed using BD FACSAria Ⅲ Cell Sorter (BD Biosciences). 2 × 10^4^ cells per sample were analyzed. Data were analyzed using FlowJo Software (Tree Star, Ashland; https://www.flowjo.com) and gated on pulse-processed propidium iodide signals to exclude doublets and large aggregates using a multiparameter gate strategy.

### Isolation of ribosomes and nuclei

Cells were cultured in cystine-containing media or cystine-deficient media for 24 h and corrected by centrifugation at 1500*g* for 3 min at 4 °C. For the isolation of ribosomes, the cells were resuspended in 600 μl lysis buffer (20 mM Tris–HCl pH 7.5, 150 mM NaCl, 5 mM MgCl_2_, 1 mM DTT and 1% Triton-X 100). After the addition of 7.5 μl of 2 U/μl DNase I, the lysates were kept on ice for 15 min, and then centrifuged at 20,000*g* for 10 min at 4 °C. The supernatants (300 μl) were layered over 900 μl sucrose cushion (20 mM Tris–HCl pH 7.5, 150 mM NaCl, 5 mM MgCl_2_, 1 mM DTT, and 1 M sucrose) in the tube and centrifugated in the TLA-120.2 Fixed-Angle Rotor (Beckman Coulter) at 100,000*g* for 1 h at 4 °C. Then, total RNA and proteins were extracted from the supernatants using ReliaPrep RNA Miniprep System (Promega) and CelLytic MT (Sigma-Aldrich), respectively. For the isolation of nuclei, cells were resuspended in 300 μl nuclear isolation buffer (30 mM Hepes-HCl pH 7.9, 30 mM KCl, 0.3 mM EGTA, 0.3 mM EDTA, 3 mM DTT, PMSF, aprotinin, and leupeptin) and kept on ice for 15 min. After the addition of 18.75 μl of 10% Nonidet P-40, the lysates were centrifugated at 500*g* for 3 min at 4 °C. The resulting pellet was washed with nuclear isolation buffer twice. Then, total RNA and proteins were extracted from the supernatants using ReliaPrep RNA Miniprep System and nucleus protein isolation buffer (20 mM Hepes-HCl pH 7.9, 400 mM NaCl, 1 mM EGTA, 1 mM EDTA, 1 mM DTT, PMSF, aprotinin, and leupeptin), respectively.

### Western blotting

The total protein of the cells was prepared using CelLytic MT according to the manufacturer’s instructions. Membrane fractions were prepared from cultured cells using a Fraction-PREP Cell Fractionation Kit (Abcam) according to the manufacturer’s instructions. Denatured samples were separated by SDS-PAGE and then transferred onto polyvinylidene difluoride membranes. The membranes were incubated with primary antibodies against *Ccnd1* (RM-9104, Thermo Fisher Scientific, RRID: AB_149914), *Ccnd2* (sc-181, Santa Cruz Biotechnology, RRID: AB_2244051), *Ccnd3* (sc-182, Santa Cruz Biotechnology, RRID: AB_2259653), cyclin A (sc-596, Santa Cruz Biotechnology, RRID: AB_631330), cyclin B1 (sc-245, Santa Cruz Biotechnology, RRID: AB_627338), cyclin E (sc-481, Santa Cruz Biotechnology, RRID: AB_2275345), p84 (10920-1-AP; Proteintech, RRID: AB_2202239), ribosomal protein S6 (RPS6; sc-74459; Santa Cruz Biotechnology, RRID: AB_1129205), p-GCN2 (PA5-105886; Thermo Fisher Scientific, RRID: AB_2817285), GCN2 (#3302; Cell Signaling Technology, RRID: AB_2277617), 4E-BP1 (#9452, Cell Signaling Technology, RRID: AB_331692), ATF-4 (sc-200, Santa Cruz Biotechnology, RRID: AB_2058752), eIF4E (ab33768, Abcam, RRID: AB_732126), CBS (14787-1-AP, Proteintech, RRID: AB_2070970), CTH (12217-1-AP, AB_2087497), xCT (#98051, Cell Signaling Technology, RRID: AB_2800296), c-MYC (10828-1-AP, Proteintech, RRID: AB_2148585), γ-H2AX (MABI0281-20, Monoclonal Antibody Research Institute Inc), GAPDH (#5174, Cell Signaling Technology, RRID: AB_10622025), Na^+^/K^+^ ATPase (#3010, Cell Signaling Technology, RRID: AB_2060983), and β-actin (sc-1616; Santa Cruz Biotechnology, RRID: AB_630836). Specific antigen–antibody complexes were visualized using horseradish-peroxidase–conjugated anti-rabbit antibodies (ab97051, Abcam, RRID: AB_2202239) against *Ccnd1*, *Ccnd2*, *Ccnd3*, cyclin E, cyclin A, p84, p-GCN2, GCN2, 4E-BP1, ATF4, eIF4E, CBS, CTH, xCT, c-MYC, GAPDH, and Na^+^/K^+^ ATPase or anti-mouse antibodies (sc-2005, Santa Cruz Biotechnology, RRID: AB_631736) against cyclin B1, RPS6, and γ-H2AX and ImmunoStar LD (Fujifilm Wako Pure Chemical). The membranes were photographed, and the density of each band was analyzed using ImageQuant LAS4010 (GE Healthcare Life Sciences, Buckinghamshire, UK; https//www.gehealthcare.com) and ImageJ software (https://imagej.net).

### Quantitative RT-PCR analysis

Total RNA was extracted using RNA iso plus (Takara Bio Inc) according to the manufacturer’s instructions. Complementary DNA was synthesized by reverse transcription using a ReverTra Ace qPCR RT Kit (Toyobo, Osaka, Japan). Real-time PCR analysis was performed on diluted complementary DNA samples using THUNDERBIRD SYBR qPCR Mix (Toyobo) with the LightCycler 96 system (Roche Diagnostics). Data were normalized using β-actin mRNAs. Sequences of the primers are listed in Table 1.

**Table 1:** Primer sets for quantitative RT-PCR analysis

Gene	Primer sequence
Mouse	Forward 5′-ACTGTCGAGTCGCGTCC-3′
*Actin*	Reverse 5′-CGCAGCGATATCGTCATCCAT-3′
Mouse	Forward 5′-ATTCCCTTGACTGCCGAGAA-3′
*Ccnd1*	Reverse 5′-GCCAGGTTCCACTTGAGCTT-3′
Mouse	Forward 5′-GAGTGGGAACTGGTAGTGTTG-3′
*Ccnd2*	Reverse 5′-CGCACAGAGCGATGAAGGT-3′
Mouse	Forward 5′-CAGACCGTAACCATTATAGACGC-3′
*Yb-1*	Reverse 5′-ATCCCTCGTTCTTTTCCCCAC-3′
Mouse	Forward 5′-CTGTGGATTTTCGGGTACGG-3′
*Chac1*	Reverse 5′-CTCGGCCAGGCATCTTGTC-3′
Mouse	Forward 5′-GGGGAGTCTGGAACATTGTGAA-3′
*Ptgs2*	Reverse 5′-GTCACATTGTAAGTAGGTGGACT-3′

### Plasmid construction

The upstream region (from bp −990 to +57, where +1 indicates the transcription start site) of the mouse *Ccnd1* gene (AF212040) and the upstream region (from bp −962 to +147, where +1 indicates the transcription start site) of the mouse *Ccnd2* gene (AF015788) were ampliﬁed using Platinum PCR SuperMix High Fidelity (Invitrogen Life Technologies). These sequences were subcloned into the pGL3 luciferase reporter vector (Promega) using KpnI and NheI restriction sites.

cDNA was synthesized from BNL 1ME A.7 R.1 cells as described above. The coding sequence regions of the mouse *Ccnd1* or *Ccnd2* genes were amplified using PrimeSTAR MAX DNA polymerase (Takara Bio Inc). These sequences were cloned into the pLVSIN-CMV-pur vector (Takara Bio Inc) using EcoRI and BamHI restriction sites for *Ccnd1* and using EcoRI and XbaI restriction sites for *Ccnd2*. As the *Eif2ak4* (coding GCN2) expressing vector, pMXs-mGCN2-FLAG-IP was a gift from Seiichi Oyadomari (Addgene plasmid #101794, RRID: Addgene_101794) (62). The coding sequence region of this vector was subcloned into the pLVSIN-CMV-pur vector using XbaI and NotI restriction sites. Primer sets for plasmid construction are listed in Table 2.

**Table 2:** Primer sets for plasmid construction

Gene	Primer sequence
Mouse	Forward 5′-ATAGAATTCATGGAACACCAGCTCCTGT-3′
*Ccnd1*	Reverse 5′-ATAGGATCCTCAGATGTCCACATCTCGCA-3′
Mouse	Forward 5′-ATAGAATTCATGGAGCTGCTGTGC-3′
*Ccnd2*	Reverse 5′-ATATCTAGATCACAGGTCAACATCCCG-3′
Mouse	Forward 5′-AGATCTAGAGCCACCATGGCTGGGGGC-3′
*Eif2ak4*	Reverse 5′-ATAGCGGCCGCCTACTTGTCATCGTCATCCT-3′

### Luciferase reporter assay

BNL 1ME A.7 R.1 cells were seeded at a density of 5.0 × 10^4^ cells per well in 24-well plates. Lipofectamine LTX regent (Invitrogen Life Technologies) was used for transfection with 50 ng of the pGL3 reporter constructs. Additionally, 1 ng of the pRL-TK vector (Promega) was transfected as an internal control reporter. Cells were harvested 24 h after transfection, and culture media were changed to cystine-containing media or cystine-deficient media. After 24 h of incubation, lysates were analyzed using the Dual-Luciferase reporter assay system (Promega) with a GloMax 20/20 Luminometer (Promega). The ratio of ﬁreﬂy (derived from pGL3 reporter constructs) to Renilla (derived from pRL-TK) luciferase activity in each sample served as a measure of the normalized luciferase activity.

### Co-immunoprecipitation analysis

BNL 1 ME A.7 R.1 cells were homogenized in coimmunoprecipitation (Co-IP) buffer (50 mM Tris–HCl (pH 7.4), 150 mM NaCl, 0.5% NP-40, and 5% glycerol). After 10 min of incubation on ice, homogenates were centrifuged for 10 min at 1500*g* and 4 °C. Dynabeads Protein G beads (Invitrogen) bound with 2 μg of anti-eIF4E antibody (sc-271480, Santa Cruz Biotechnology, RRID: AB_10649368) or anti-mouse IgG antibody (140–09511, Fujifilm Wako Pure Chemical) were added to the supernatant and incubated for 2 h at 4 °C. After washing two times with Co-IP buffer, the reactants were eluted using 50 mM glycine (pH 2.8). Co-IP lysates were denatured at 95 °C for 5 min with 50 μM Tris–HCl, 2% SDS, 0.06% 2-mercaptoethanol, and 1% glycerol.

### Construction of *Eif4ebp1*-knockdown cells

siRNA against the mouse *Eif4ebp1* gene was purchased from Sigma-Aldrich. BNL 1ME A.7 R.1 cells were transfected with 100 pmol *Eif4ebp1* siRNA or siRNA Universal Negative Control#1 (SIC001, Sigma-Aldrich) using Lipofectamine 2000 (Thermo Fisher Scientific). Downregulation of each gene was confirmed by Western blotting.

### Animals and treatments

We housed six to eight male BALB/c mice per cage, with each cage being 30 cm × 40 cm × 16 cm, providing enough space. They were kept under a standardized light-dark cycle at 24 °C ± 1 °C, humidity at 60 ± 10% and food and water provided *ad libitum*. BNL 1ME A.7 R.1 cells (2.8 × 10^6^ cells) suspended in 70 μl of PBS were implanted subcutaneously into the back of male BALB/c mice. After confirming the tumor formation, drug administration was initiated. BNL 1ME A.7 R.1-implanted mice were intraperitoneally injected with erastin (30 mg/kg body weight) or vehicle (10% dimethyl sulfoxide and 50% PEG in saline) for every 3 days. Day 15 after the initiation of erastin administration, mice were sacrificed and protein expression levels of D-type cyclin in tumor were measured by Western blotting. All protocols involving mice were reviewed and approved by the Animal Care and Use Committee of Kyushu University (A22-329-1). All methods were performed according to the relevant guidelines and regulations.

### Construction of D-type cyclins or *Eif2ak4* overexpressing cells

The lentivirus vector expressing *Ccnd1*, *Ccnd2*, or *Eif2ak4* expressing vectors were prepared and constructed as described above. Lentivirus particles were prepared by the Lentiviral High Titer Packaging Mix with pLVSIN series (Clontech) using Lenti-X 293 T-cell lines. To select clones stably expressing vectors, cells were maintained in a medium containing 5 μg/ml of puromycin (Fujifilm Wako Pure Chemical). The expressions of *Ccnd1*, *Ccnd2*, and GCN2 were confirmed by Western blotting. To construct control cells, BNL 1ME A.7 R.1 cells were infected with lentivirus particles derived from the pLVSIN-CMV-pur vector and were cultured in puromycin-containing medium, as described above, to select the stably expressing cells.

### Statistical analysis

All statistical analyses were conducted using JMP pro17.0 (SAS Institute Japan, Tokyo, Japan; https://www.jmp.com/ja_jp/home.html). All data were checked the normality and equal variances before performing statistical analysis. The significance of differences among groups was analyzed by one-way or two-way ANOVA, followed by Tukey–Kramer’s *post hoc* test. Student’s *t* test was used for the comparison of data between two groups. A 5% level of probability was considered significant.

## Data availability

All data supporting the results of the present study are included in the article.

## Supporting information

This article contains supporting information.

## Conflict of interest

The authors declare that they have no conflicts of interest with the contents of this article.
