# Supplementary material for: Oncogenic accumulation of cysteine promotes cancer cell proliferation by regulating the translation of D-type cyclins[image]
Source: J Biol Chem. 2024 Oct 15;300(11):107890. doi: 10.1016/j.jbc.2024.107890 (PMC11650723; doi:10.1016/j.jbc.2024.107890)
Supplement: Supplementary Data [file mmc1.pdf]

Supplementary Data

Oncogenic accumulation of cysteine promotes cancer cell proliferation by regulating the translation of D-type cyclins

Running title: Pathological significance of oncogenic cysteine accumulation

*Yumi Okano[1], *Tomoaki Yamauchi[1], Runa Fukuzaki[1], Akito Tsuruta[1], Yuya Yoshida[2], Yuya Tsurudome[3], Kentaro Ushijima[3], Naoya Matsunaga[2], Satoru Koyanagi[1], Shigehiro Ohdo[1]

Supplementary Figure S1 Differential growth ability of murine cancer cells depending on amino acids.

Supplementary Figure S2 Negligible effect of extracellular cystine deprivation on the viability of primary hepatocytes.

Supplementary Figure S3 Cysteine-dependent expression of Cyclin D1 and D2 proteins in BNL 1ME A.7 R.1 cells.

Supplementary Figure S4 Effect of cysteine deprivation on the expression of Cyclin D1 and D2 in murine cancer cells.

Supplementary Figure S5 Restoring D-type cyclins can rescue the arrested cell cycle caused by cysteine deprivation.

Supplementary Figure S6 Effect of overexpression of GCN2 on the growth ability and GCN2 / ATF4 / 4E-BP1 pathway in BNL 1ME A.7 R.1 cells.

Supplementary Figure S7 The protein levels of Cyclin D1 and D2 in 4T1, RenCa, and Colon-26 cells after treatment with erastin.

Supplementary Figure S8 The protein levels of cysteine synthetic enzymes and cystine uptake transporters in primary hepatocytes and murine cancer cells.

Supplementary Figure S9 Involvement of YB-1 in the transcriptional regulation of the mouse *Ccnd1* and *Ccnd2* genes.

Supplementary Figure S10 Downregulation of c-MYC expression in BNL 1ME A.7 R.1 cells under cysteine depletion conditions.

Supplementary Figure S11 The ability of ferrostatin-1 to attenuate the cytotoxic effect of erastin on BNL 1ME A.7 R.1 cells.

Supplementary Figure S12 Unedited full blots of Figure 2D

Supplementary Figure S13 Unedited full blots of Figure 2E

Supplementary Figure S14 Unedited full blots of Figure 3C

Supplementary Figure S15 Unedited full blots of Figure 4B

Supplementary Figure S16 Unedited full blots of Figure 4C

Supplementary Figure S17 Unedited full blots of Figure 4D

Supplementary Figure S18 Unedited full blots of Figure 5C

Supplementary Figure S19 Unedited full blots of Figure 5D

Supplementary Figure S20 Unedited full blots of Supplementary Figure S2B

Supplementary Figure S21 Unedited full blots of Supplementary Figure S3

Supplementary Figure S22 Unedited full blots of Supplementary Figure S4

Supplementary Figure S23 Unedited full blots of Supplementary Figure S5A and S5B

Supplementary Figure S24 Unedited full blots of Supplementary Figure S6B

Supplementary Figure S25 Unedited full blots of Supplementary Figure S7

Supplementary Figure S26 Unedited full blots of Supplementary Figure S8A

Supplementary Figure S27 Unedited full blots of Supplementary Figure S8B

Supplementary Figure S28 Unedited full blots of Supplementary Figure S8C

Supplementary Figure S29 Unedited full blots of Supplementary Figure S10

Supplementary Figure S30 Unedited full blots of Supplementary Figure S11D

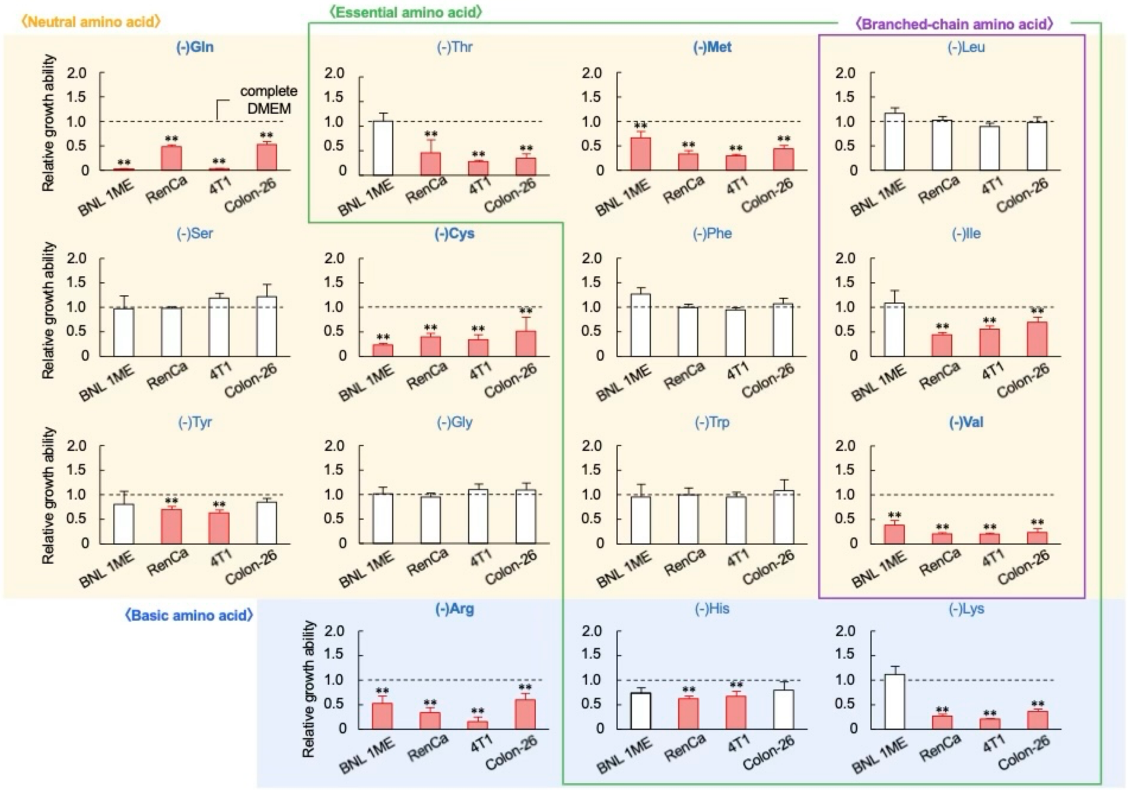

Supplementary Figure S1 Differential growth ability of murine cancer cells depending on amino acids. Four types of murine cancer cells (BNL 1ME A.7 R.1 hepatocarcinoma, 4T1 breast cancer, RenCa renal adenocarcinoma, and Colon-26 adenocarcinoma) were incubated for 48 h in single amino acid-deficient media by removing each of the 15 amino acids from the culture media, and their growth was measured following media replacement. The basal cell viability (0 h) was set to 1.0. Each value represents the mean with S.D. (n = 5). **; *P* < 0.01, significant difference from basal cell viability (Student's *t*-test).

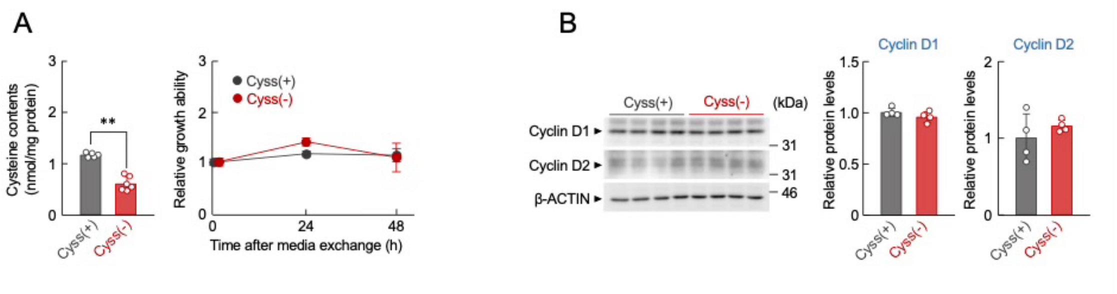

Supplementary Figure S2 Negligible effect of extracellular cystine deprivation on the viability of primary hepatocytes. A, Primary hepatocytes were incubated in cystine-containing (Cyss (+)) or cystine-deficient (Cyss (-)) media. Left column shows intracellular cysteine levels 16 h after incubation in Cyss (+) and Cyss (-). Right line graph shows the cell viability during incubation in Cyss (+) and Cyss (-). The basal cell viability of the Cyss (+) media group (0 h) was set at 1.0. Each value represents the mean with S.D. (n = 4–6). **; $P < 0.01$, significant difference between the two groups ($t_9 = 8.228$, $P < 0.001$; Student's *t*-test). **B,** The protein levels of Cyclin D1 and D2 in primary hepatocytes 24 h after incubation in Cyss (+) or Cyss (-) media. The protein levels were normalized to those of β-ACTIN. Each value represents the mean with S.D. (n = 4).

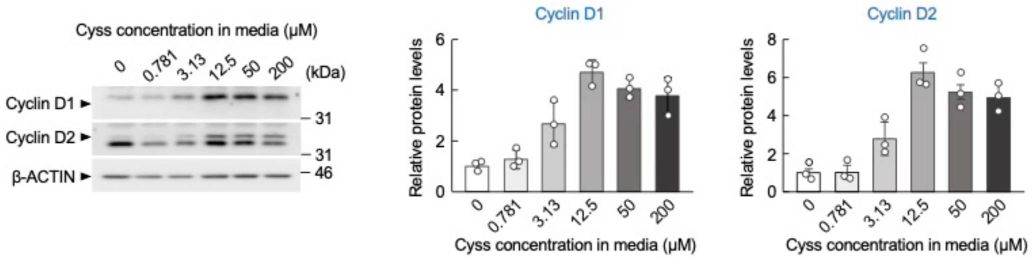

Supplementary Figure S3 Cysteine-dependent expression of Cyclin D1 and D2 proteins in BNL 1ME A.7 R.1 cells. As shown in Fig. 2A, the intracellular contents of cysteine in BNL 1ME A.7 R.1 cells increased in an extracellular cysteine concentration-dependent manner. Cells were incubated in conditional media containing indicated concentrations of cystine for 24 h. Each value represents the mean with S.D. (n = 3).

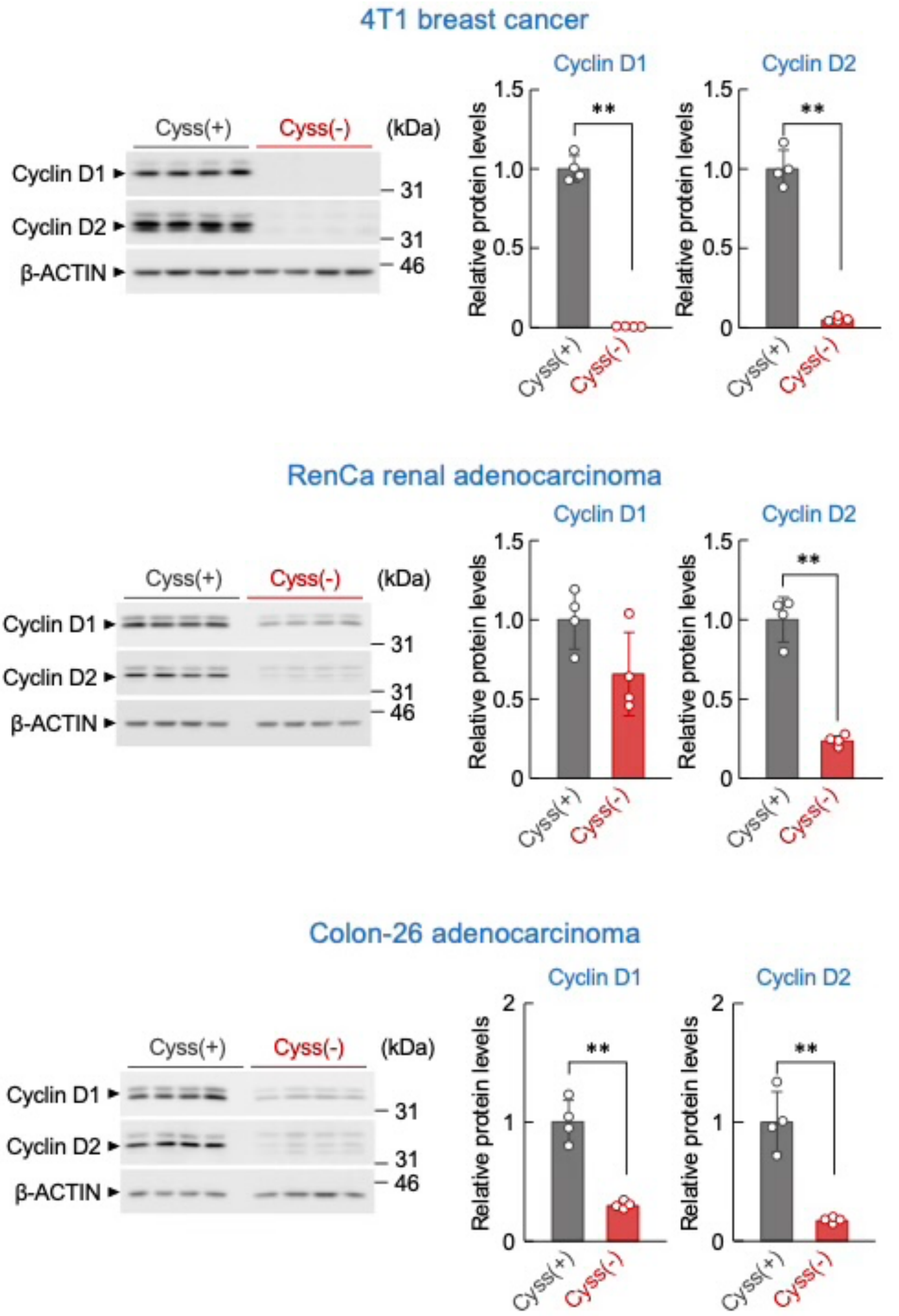

Supplementary Figure S4 Effect of cysteine deprivation on the expression of Cyclin D1 and D2 in murine cancer cells. 4T1 cells, RenCa cells, and Colon-26 cells were incubated in Cyss (+) or Cyss (-) media for 24 h. The protein levels of Cyclin D1 and D2 were normalized to those of β-ACTIN. Each value represents the mean with S.D. (n = 4). **; $P < 0.01$, significant difference between the two groups ($t_6 = 25.400$, $P < 0.001$ for Cyclin D1 of 4T1 cells; $t_6 = 16.135$, $P < 0.001$ for Cyclin D2 of 4T1 cells; $t_6 = 10.454$, $P < 0.001$ for Cyclin D2 of RenCa cells; $t_6 = 7.632$, $P < 0.001$ for Cyclin D1 of Colon-26 cells; $t_6 = 6.482$, $P < 0.001$ for Cyclin D2 of Colon-26 cells; Student's *t*-test).

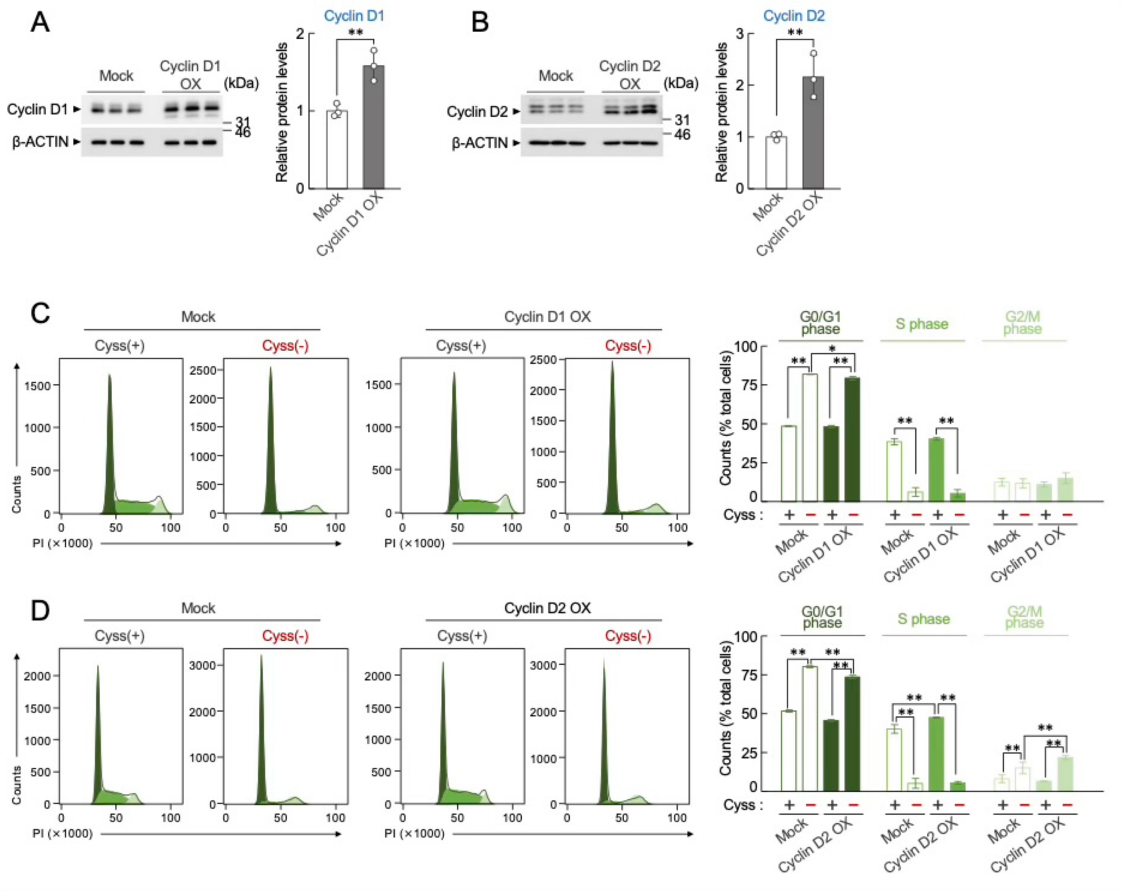

Supplementary Figure S5 Restoring D-type cyclins can rescue the arrested cell cycle caused by cysteine deprivation. A and **B,** The protein levels of Cyclin D1 (A) and Cyclin D2 (B) in BNL 1ME A.7 R.1 cells. The protein levels were normalized to those of β-ACTIN. Each value represents the mean with S.D. (n = 3). **; $P < 0.01$, significant difference between the two groups ($t_4 = 4.710$, $P = 0.009$ for Cyclin D1; $t_4 = 4.680$, $P = 0.010$ for Cyclin D2; Student's *t*-test). **C** and **D,** Representative images (left) and quantification (right) of the cell cycle distribution in Cyclin D1 (C) or Cyclin D2 (D) overexpressing BNL 1ME A.7 R.1 cells incubated in cystine-containing (Cyss (+)) or cystine-deficient (Cyss (-)) media for 24 h. Each value represents the mean with S.D. (n = 3-4). **; $P < 0.01$, *; $P < 0.05$ significant difference between the two groups (C, $F_{3,11} = 2023.978$, $P < 0.001$ for G0/G1 phase; $F_{3,11} = 245.780$, $P < 0.001$ for S phase; ANOVA with Tukey–Kramer's post hoc test / D, $F_{3,12} = 1767.122$, $P < 0.001$ for G0/G1 phase; $F_{3,12} = 423.111$, $P < 0.001$ for S phase; $F_{3,12} = 35.918$, $P < 0.001$ for G2/M phase; ANOVA with Tukey–Kramer's post hoc test).

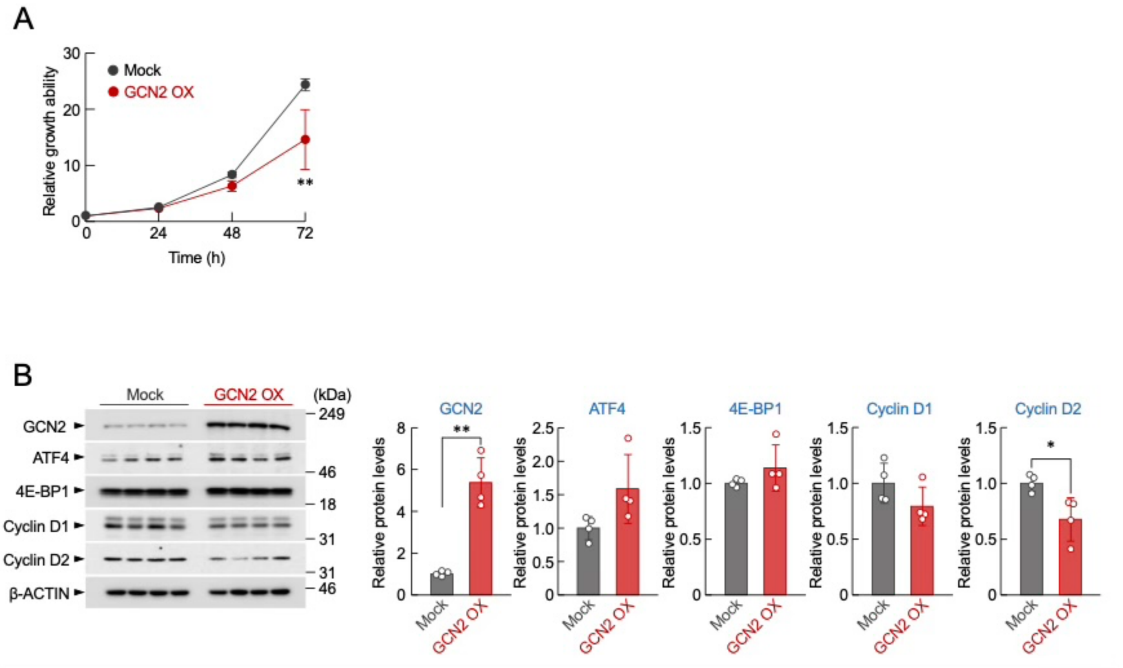

Supplementary Figure S6 Effect of overexpression of GCN2 on the growth ability and GCN2 / ATF4 / 4E-BP1 pathway in BNL 1ME A.7 R.1 cells. A, The growth ability of mock-transduced or GCN2-overexpressing (OX) BNL 1ME A.7 R.1 cells. The basal cell viability (0 h) of mock-transduced cells was set at 1.0. Each value represents the mean with S.D. (n = 6). ***P* < 0.01; significant difference between the two groups ($F_{3,40}$ = 16.495; *P* < 0.0001; Two-way ANOVA with Tukey–Kramer's post hoc test). **B,** The protein levels of GCN2, ATF4, 4E-BP1, Cyclin D1, and Cyclin D2 in mock-transduced or GCN2 OX BNL 1ME A.7 R.1 cells. The protein levels were normalized to those of β-ACTIN. Each value represents the mean with S.D. (n = 4). **; *P* < 0.01, *; *P* < 0.05 significant difference between the two groups (t_6 = 7.391, *P* < 0.001 for GCN2; t_6 = -3.140, *P* = 0.020 for Cyclin D2; Student's *t*-test).

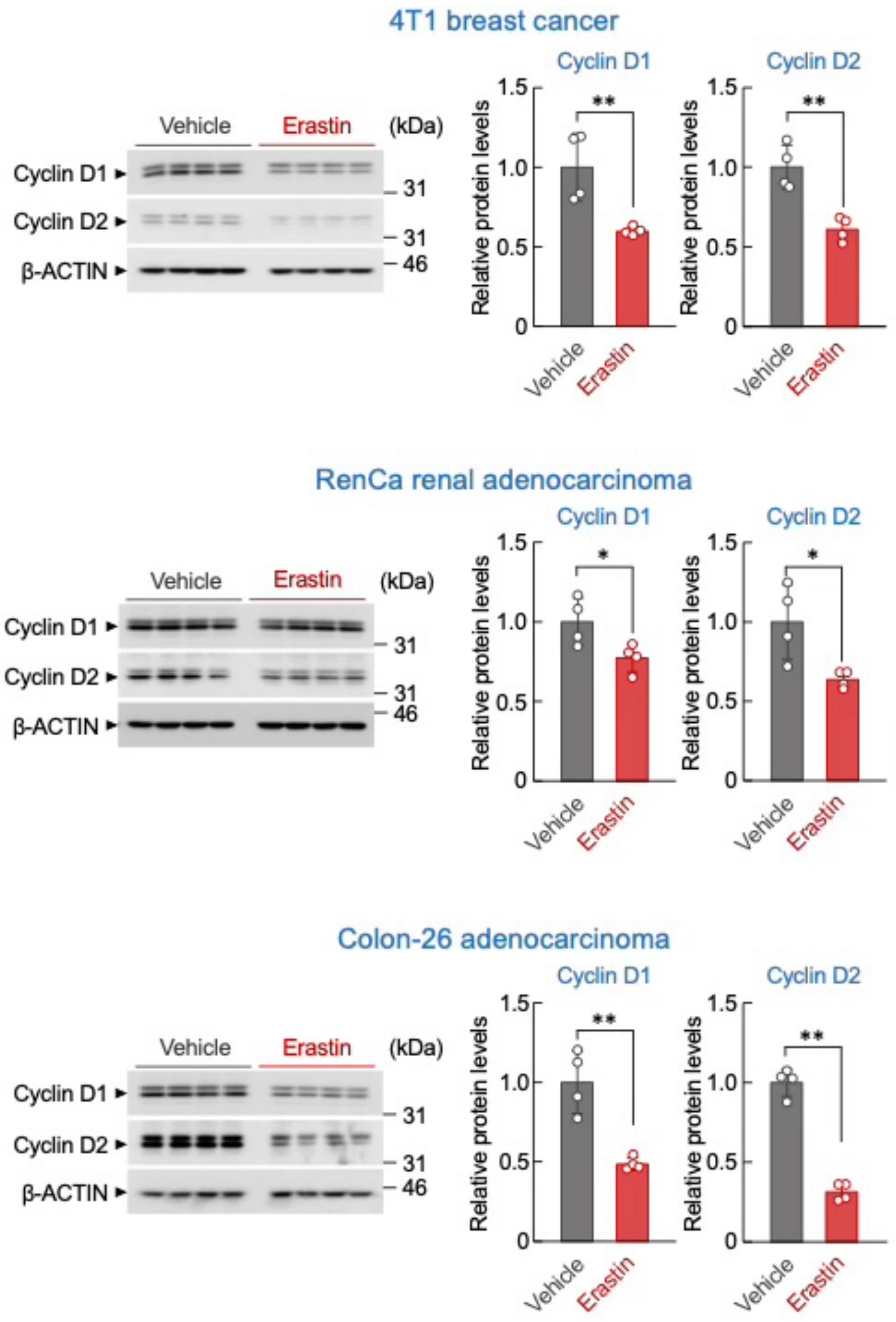

Supplementary Figure S7 The protein levels of Cyclin D1 and D2 in 4T1, RenCa, and Colon-26 cells after treatment with erastin. Cells were treated with 5 μM erastin for 24 h. The protein levels of Cyclin D1 and D2 were normalized to those of β-ACTIN. Each value represents the mean with S.D. (n = 4). **; $P < 0.01$, *; $P < 0.05$, significant difference between the two groups (t_6 = -3.739, P = 0.010 for Cyclin D1 of 4T1 cells; t_6 = -4.966, P = 0.003 for Cyclin D2 of 4T1 cells; t_6 = -2.610, P = 0.040 for Cyclin D1 of RenCa cells; t_6 = -3.012, P = 0.024 for Cyclin D2 of RenCa cells; t_6 = -5.103, P = 0.002 for Cyclin D1 of Colon-26 cells; t_6 = -13.497, P < 0.001 for Cyclin D2 of Colon-26 cells; Student's *t*-test).

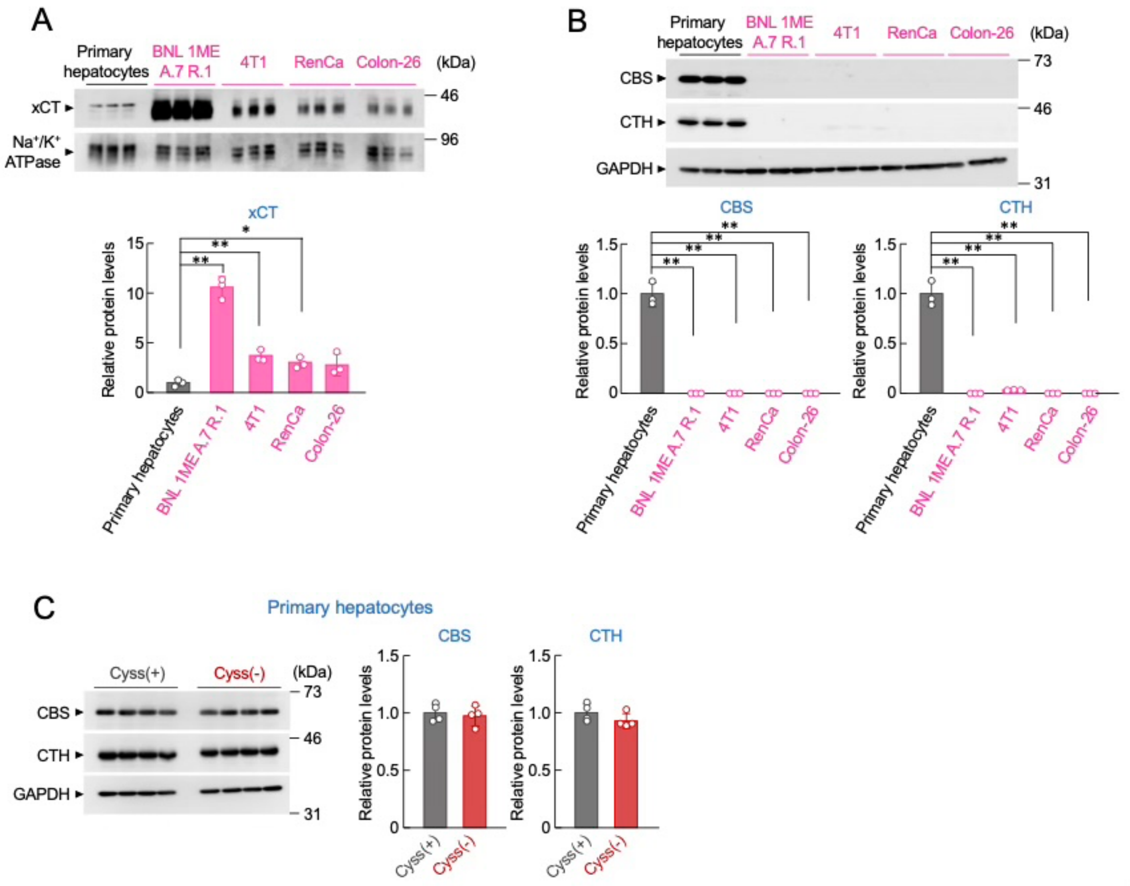

Supplementary Figure S8 The protein levels of cysteine synthetic enzymes and cystine uptake transporters in primary hepatocytes and murine cancer cells. A and **B,** The protein levels of xCT (A) cystathionine-β-synthase (CBS), and cystathionine-γ-lyase (CTH) (B) in primary hepatocytes, BNL 1ME A.7 R.1 cells, 4T1 cells, RenCa cells, and Colon-26 cells. The protein levels were normalized to those of GAPDH (for CBS and CTH) or Na^{+}/K^{+} ATPase (for xCT). Each value represents the mean with S.D. (n = 3). **; $P < 0.01$, *; $P < 0.05$, significant difference between the two groups ($F_{4,10} = 65.459$, $P < 0.001$ for xCT; $F_{4,10} = 209.016$, $P < 0.001$ for CBS; $F_{4,10} = 183.685$, $P < 0.001$ for CTH; ANOVA with Dunnett's post hoc test). **C**, The protein levels of CBS and CTH in primary hepatocytes after incubation in cystine-containing (Cyss (+)) or cystine-deficient (Cyss (-)) media for 24h. Each value represents the mean with S.D. (n = 4).

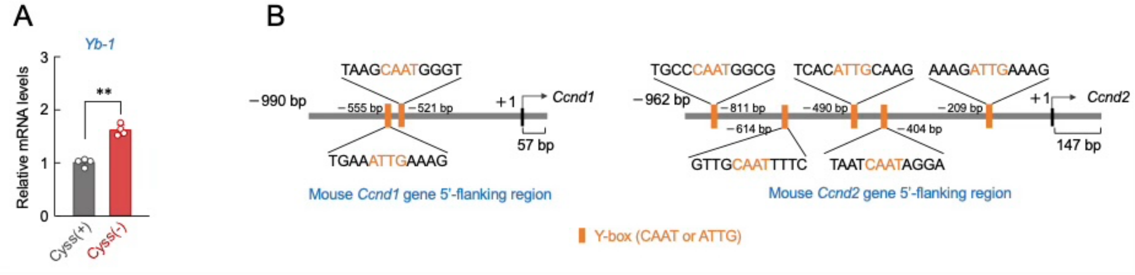

Supplementary Figure S9 Involvement of YB-1 in the transcriptional regulation of the mouse *Ccnd1* and *Ccnd2* genes. A, The mRNA levels of *Yb-1* in BNL 1ME A.7 R.1 cells after incubation in cystine-containing (Cyss (+)) or cystine-deficient (Cyss (-)) media for 24 h. The mRNA levels were normalized to those of *β-Actin*. Each value represents the mean with S.D. (n = 4). **; $P < 0.01$, significant difference between the two groups ($t_6 = 9.482$, $P < 0.001$; Student's *t*-test). **B,** Localization of Y-box in the upstream region of the mouse *Ccnd1* and *Ccnd2* genes. The numbers indicate the distance in base pairs from the putative transcription start site.

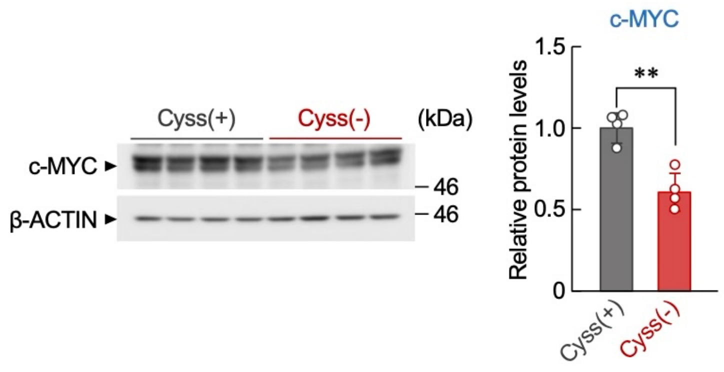

Supplementary Figure S10 Downregulation of c-MYC expression in BNL 1ME A.7 R.1 cells under cysteine depletion conditions. The protein levels of c-MYC in BNL 1ME A.7 R.1 cells after incubation in cystine-containing (Cyss (+)) or cystine-deficient (Cyss (-)) media for 24 h. The protein levels were normalized to those of β-ACTIN. Each value represents the mean with S.D. (n = 4). **; $P < 0.01$, significant difference between the two groups ($t_6 = 5.277$, $P = 0.002$; Student's *t*-test).

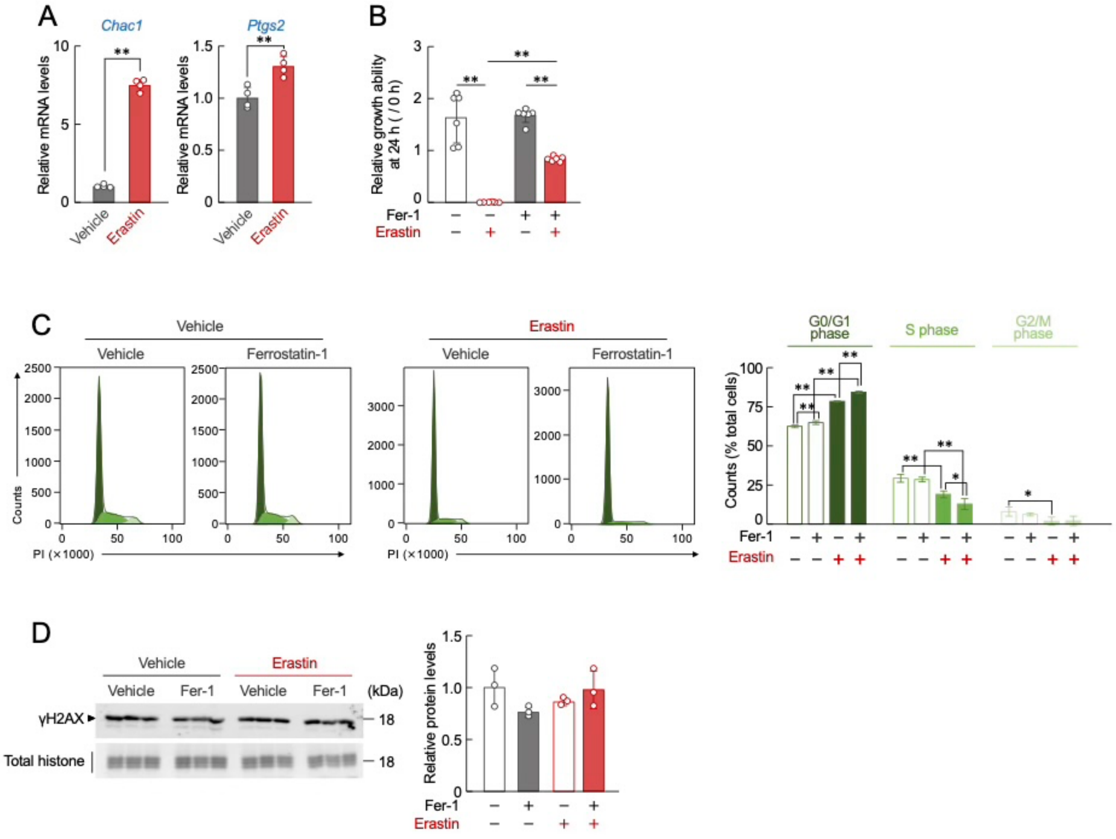

Supplementary Figure S11 The ability of ferrostatin-1 to attenuate the cytotoxic effect of erastin on BNL 1ME A.7 R.1 cells. A, The mRNA levels of ferroptosis marker, *Chac1* and *Ptgs2,* in BNL 1ME A.7 R.1 cells after treatment with vehicle or 5 μM erastin for 24 h. The mRNA levels were normalized to those of *18s*. Each value represents the mean with S.D. (n = 4). **; $P < 0.01$, significant difference between the two groups ($t_6 = 32.810$, $P < 0.001$ for *Chac1*; $t_6 = 4.160$, $P = 0.006$ for *Ptgs2*; Student's *t*-test). **B,** BNL 1ME A.7 R.1 cells were treated with 5 μM erastin in the presence or absence of 10 μM ferrostatin-1 (Fer-1) for 24 h. Each value represents the mean with S.D. (n = 6). **; $P < 0.01$, significant difference between the two groups ($F_{3,20} = 57.730$, $P < 0.001$; ANOVA with Tukey–Kramer's post hoc test). **C,** Representative images (left) and quantification (right) of the cell cycle distribution in BNL 1ME A.7 R.1 cells after treatment with 5 μM erastin or 10 μM Fer-1 for 24 h. Each value represents the mean with S.D. (n = 4). **; $P < 0.01$, significant difference between the two groups ($F_{3,12} = 609.186$, $P < 0.001$ for G0/G1 phase; $F_{3,12} = 40.077$, $P < 0.001$ for S phase; $F_{3,12} = 5.924$, $P = 0.010$ for G2/M phase; ANOVA with Tukey–Kramer's post hoc test). **D,** The protein levels of γH2AX in BNL 1ME A.7 R.1 cells after treatment with 5 μM erastin or 10 μM Fer-1 for 24 h. Each value represents the mean with S.D. (n = 3).

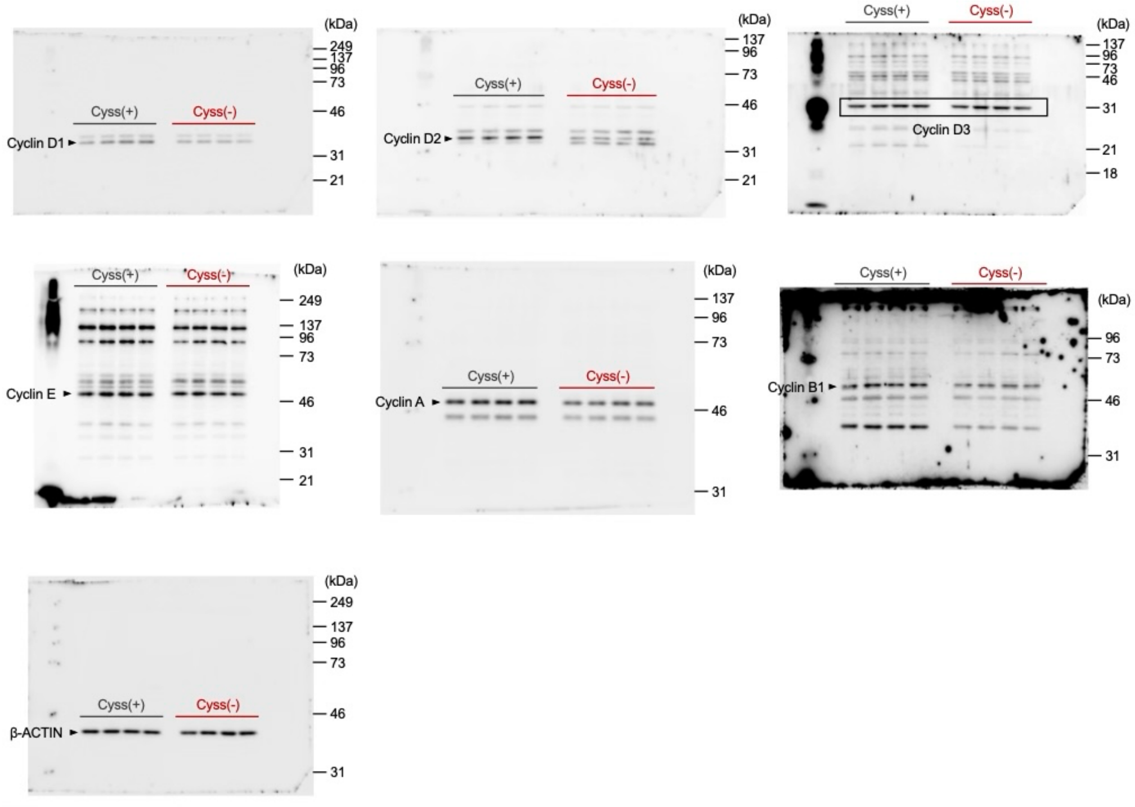

Supplementary Figure S12 Unedited full blots of Figure 2D

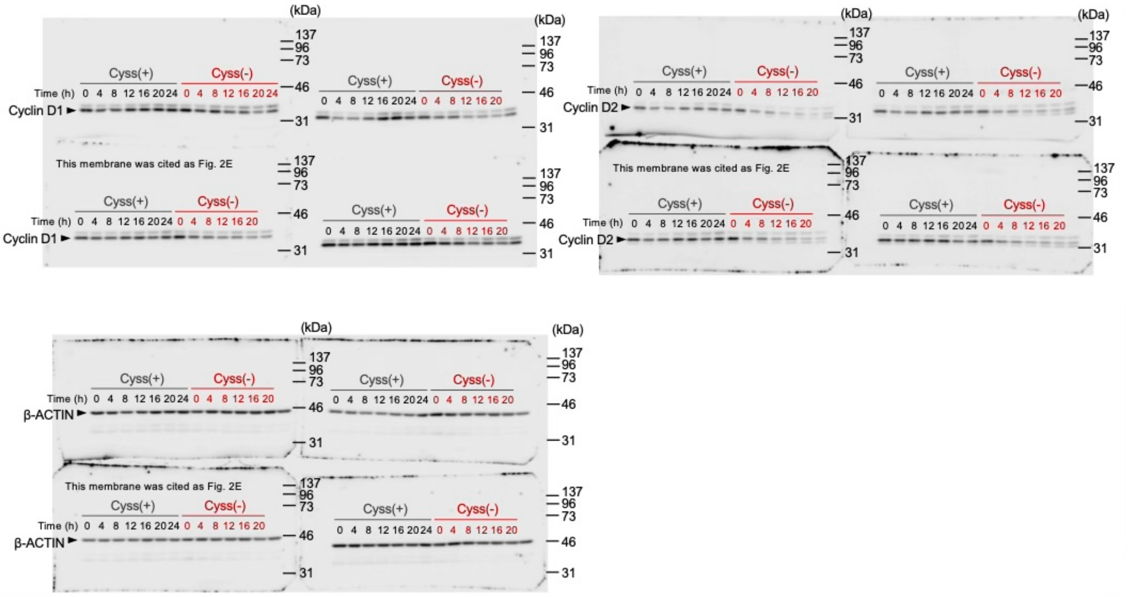

Supplementary Figure S13 Unedited full blots of Figure 2E

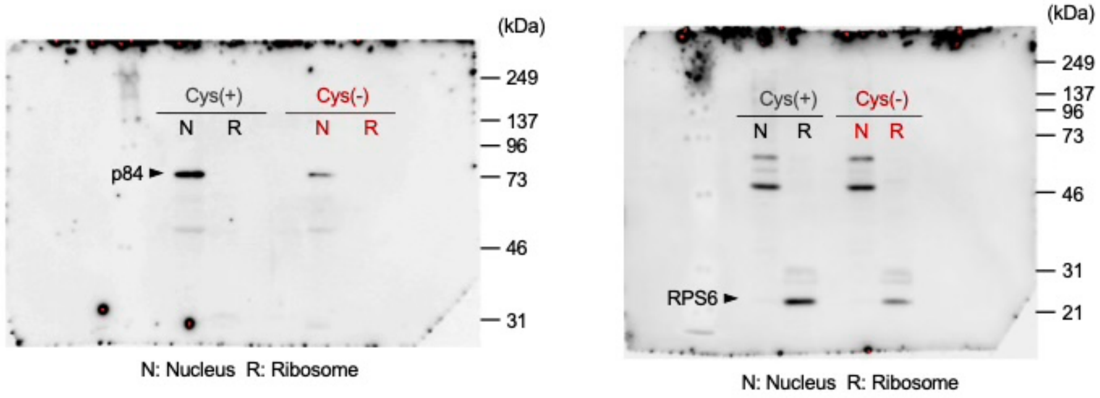

Supplementary Figure S14 Unedited full blots of Figure 3C

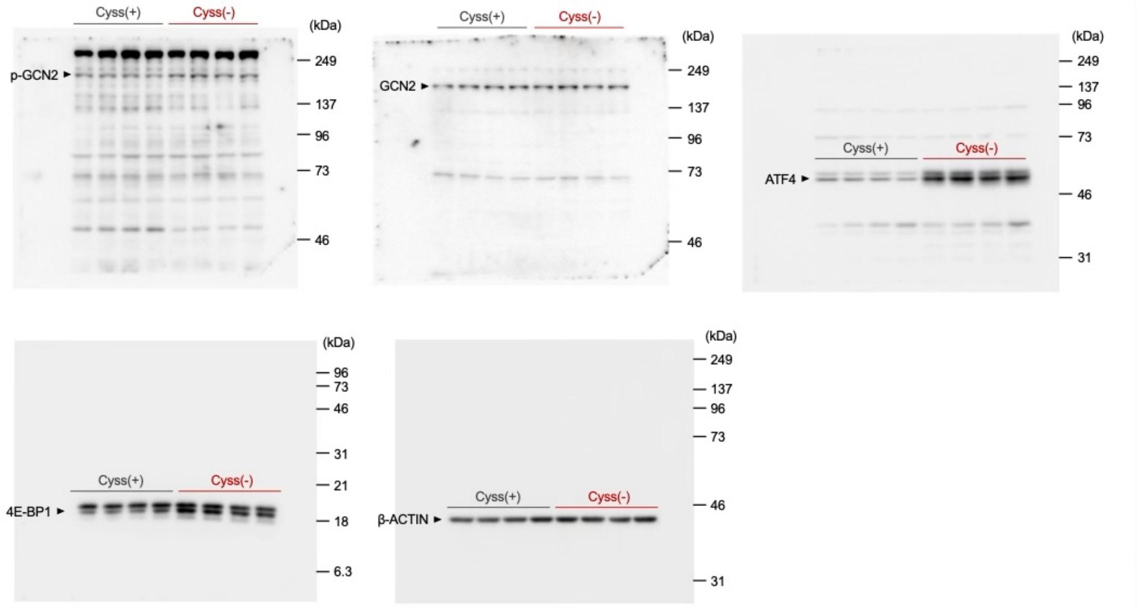

Supplementary Figure S15 Unedited full blots of Figure 4B

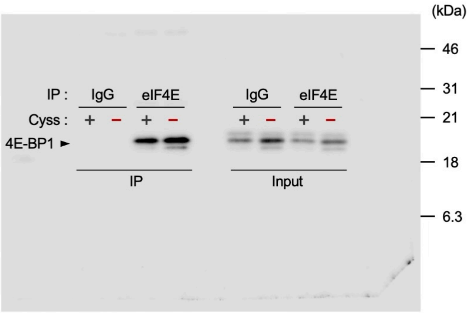

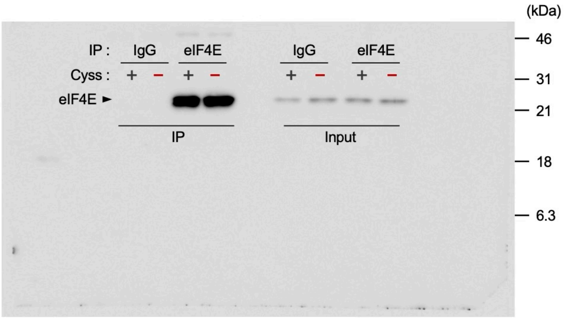

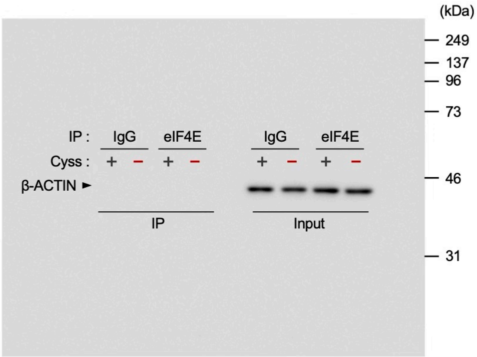

Supplementary Figure S16 Unedited full blots of Figure 4C

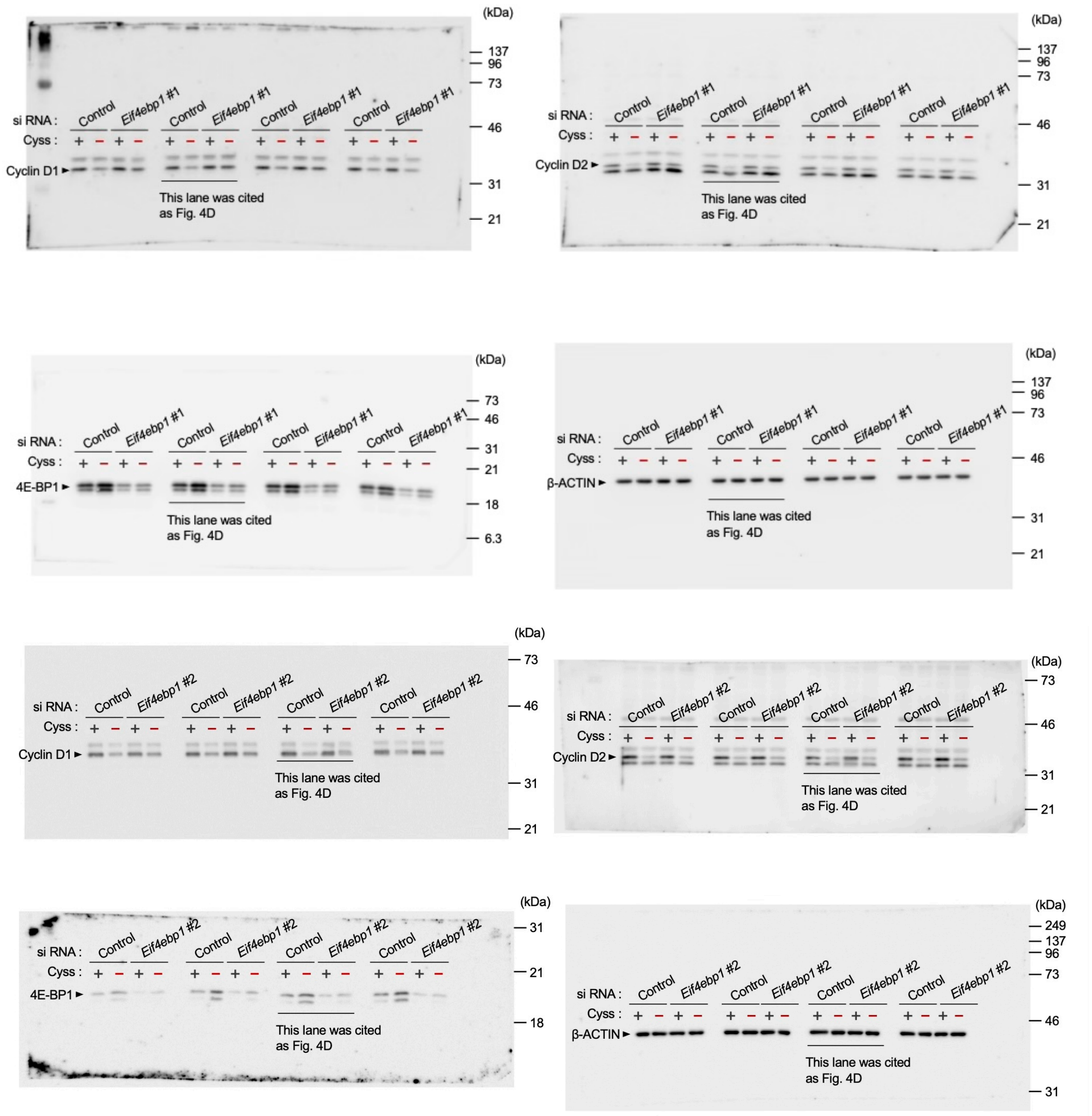

Supplementary Figure S17 Unedited full blots of Figure 4D

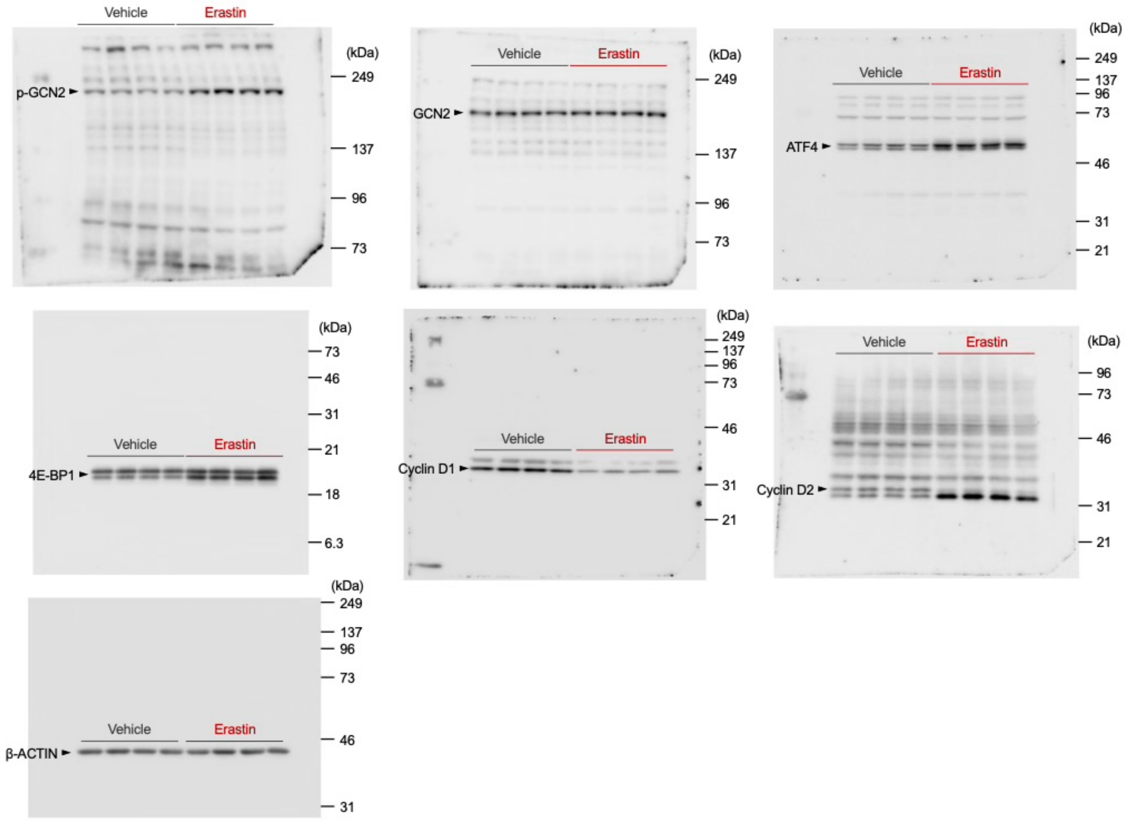

Supplementary Figure S18 Unedited full blots of Figure 5C

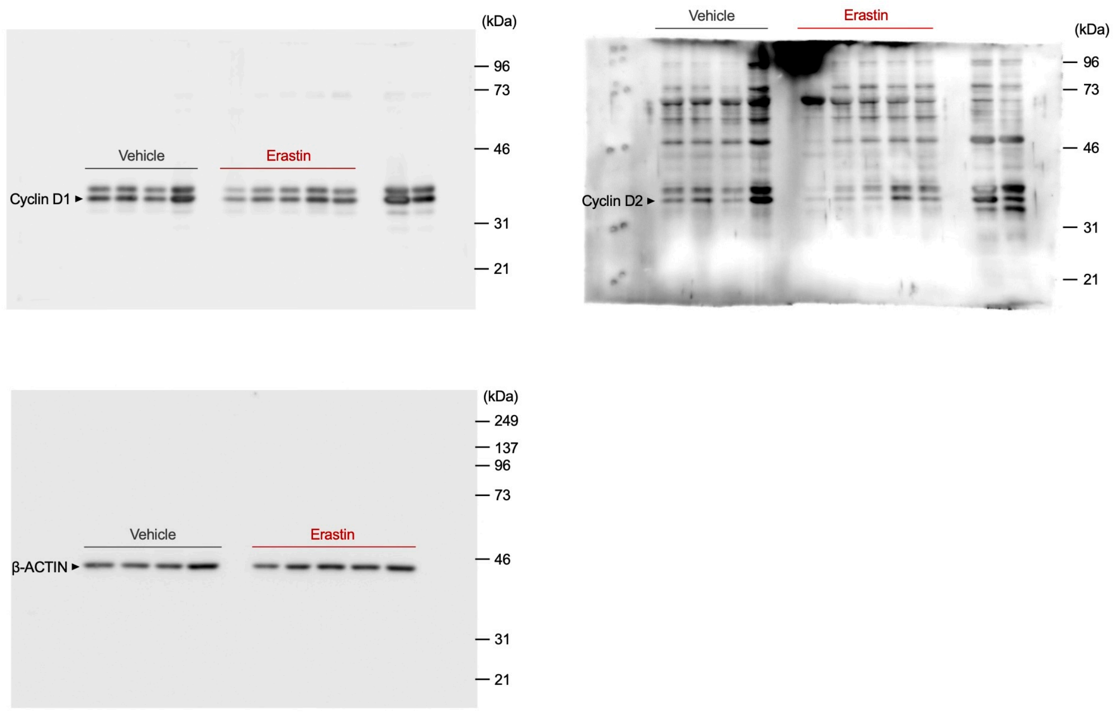

Supplementary Figure S19 Unedited full blots of Figure 5D

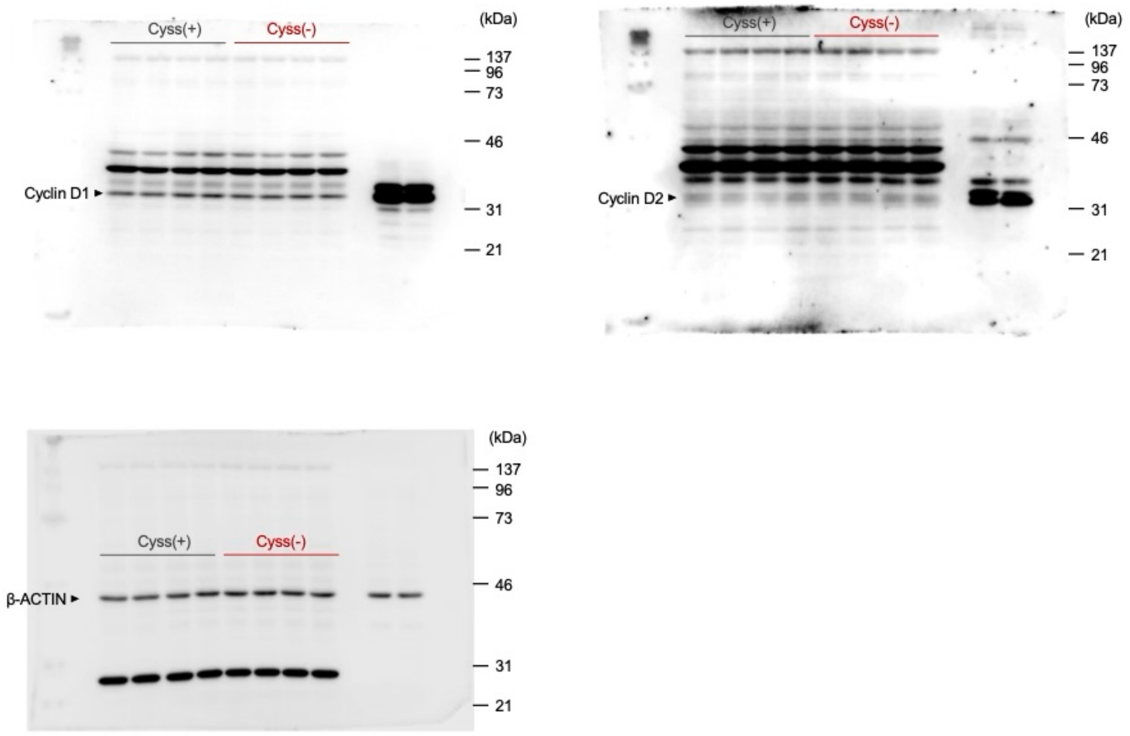

Supplementary Figure S20 Unedited full blots of Supplementary Figure S2B

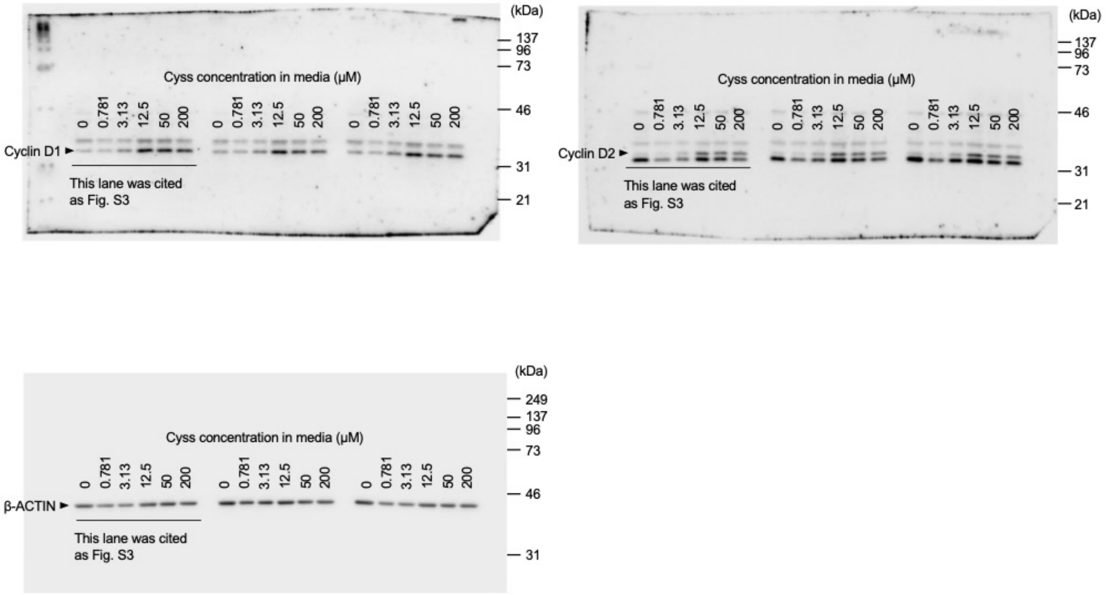

Supplementary Figure S21 Unedited full blots of Supplementary Figure S3

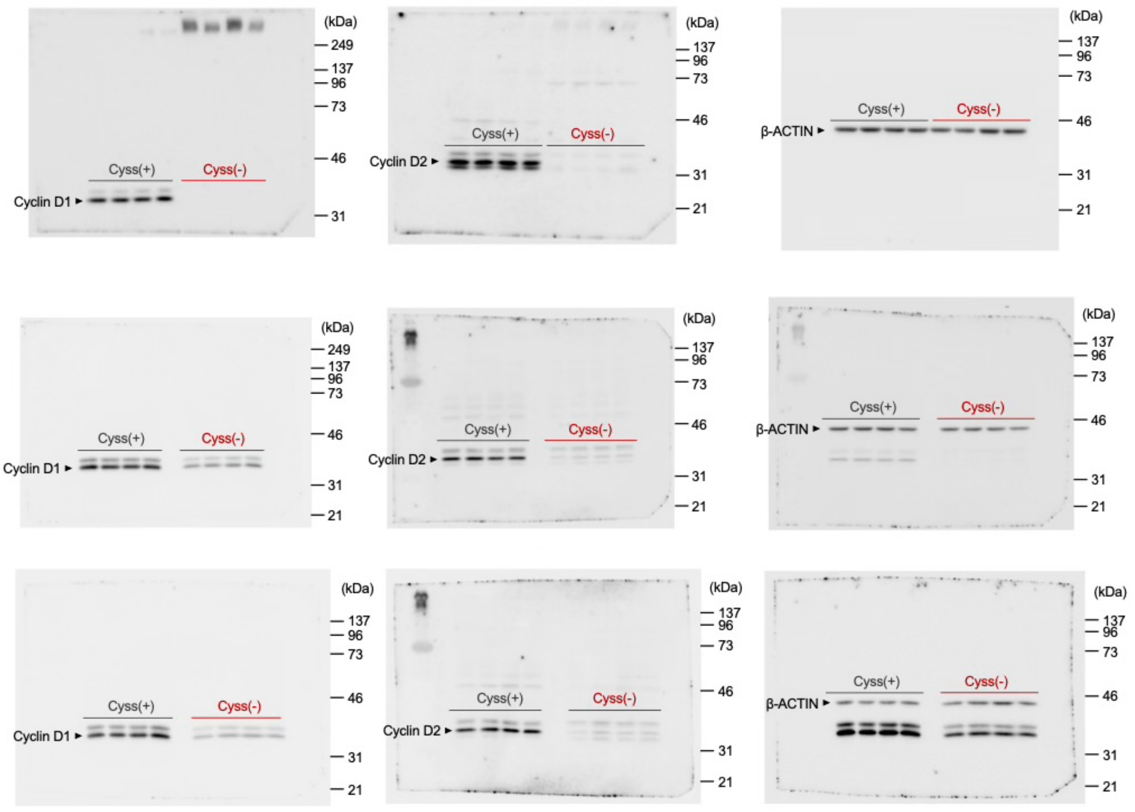

Supplementary Figure S22 Unedited full blots of Supplementary Figure S4

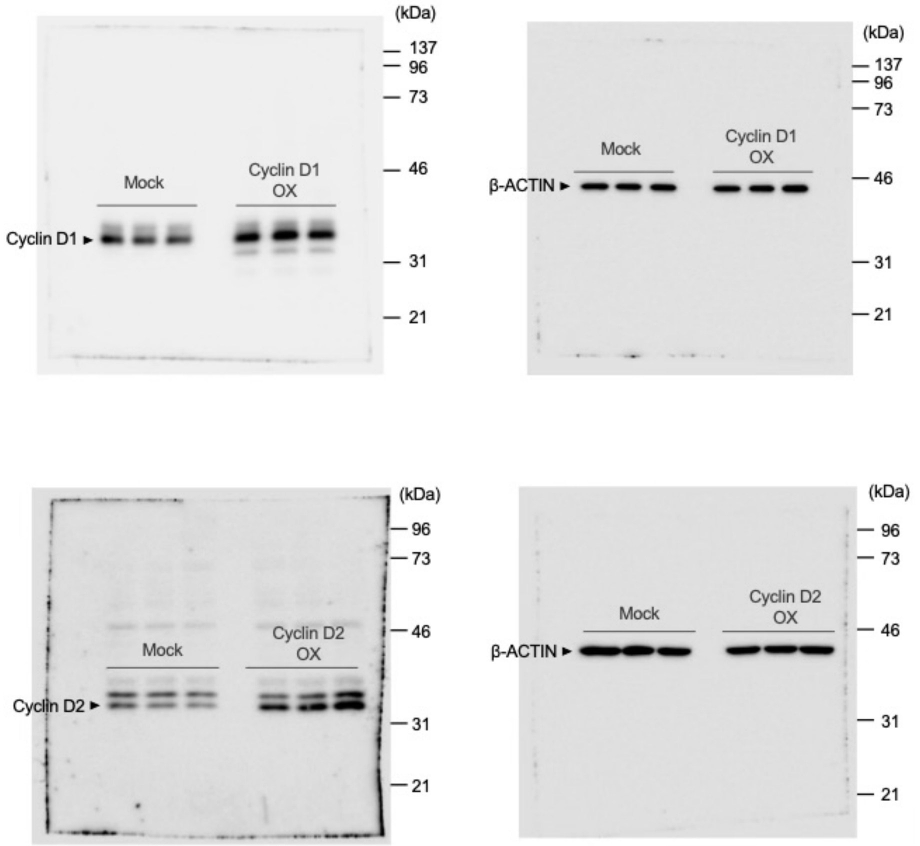

Supplementary Figure S23 Unedited full blots of Supplementary Figure S5A and S5B

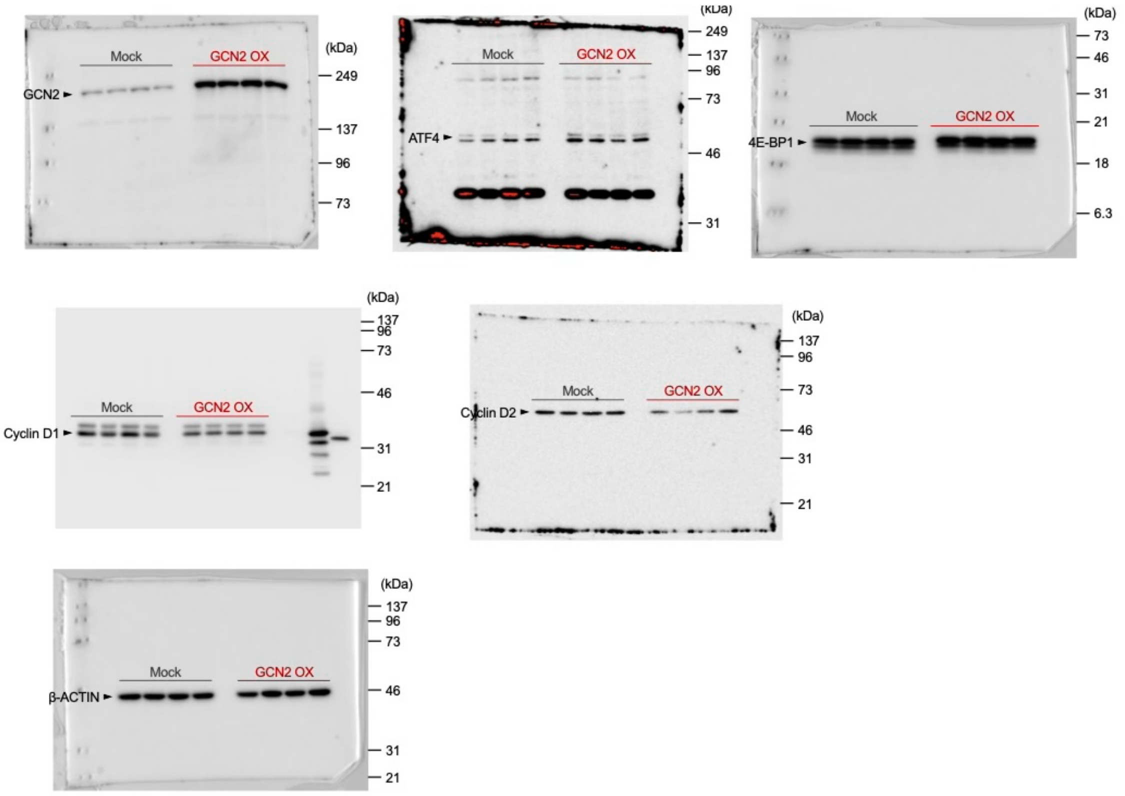

Supplementary Figure S24 Unedited full blots of Supplementary Figure S6B

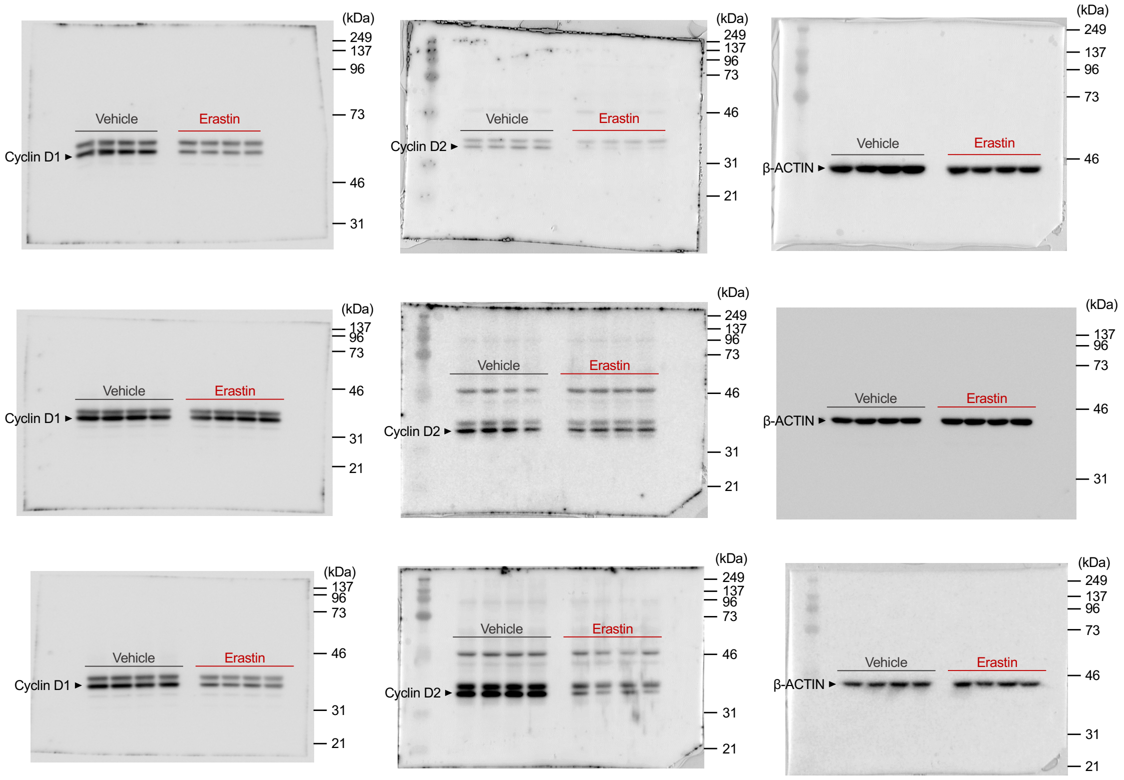

Supplementary Figure S25 Unedited full blots of Supplementary Figure S7

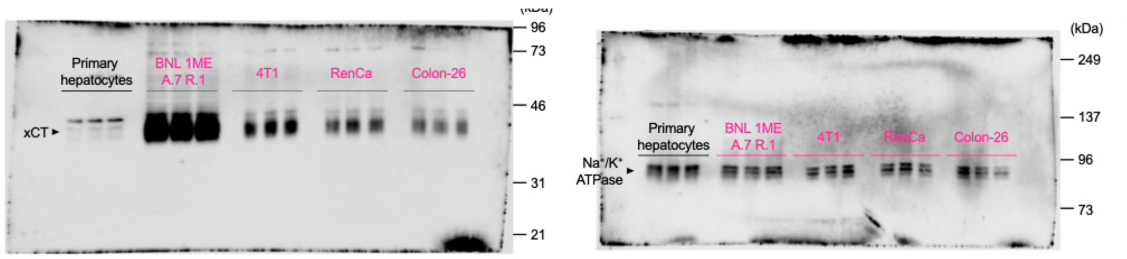

Supplementary Figure S26 Unedited full blots of Supplementary Figure S8A

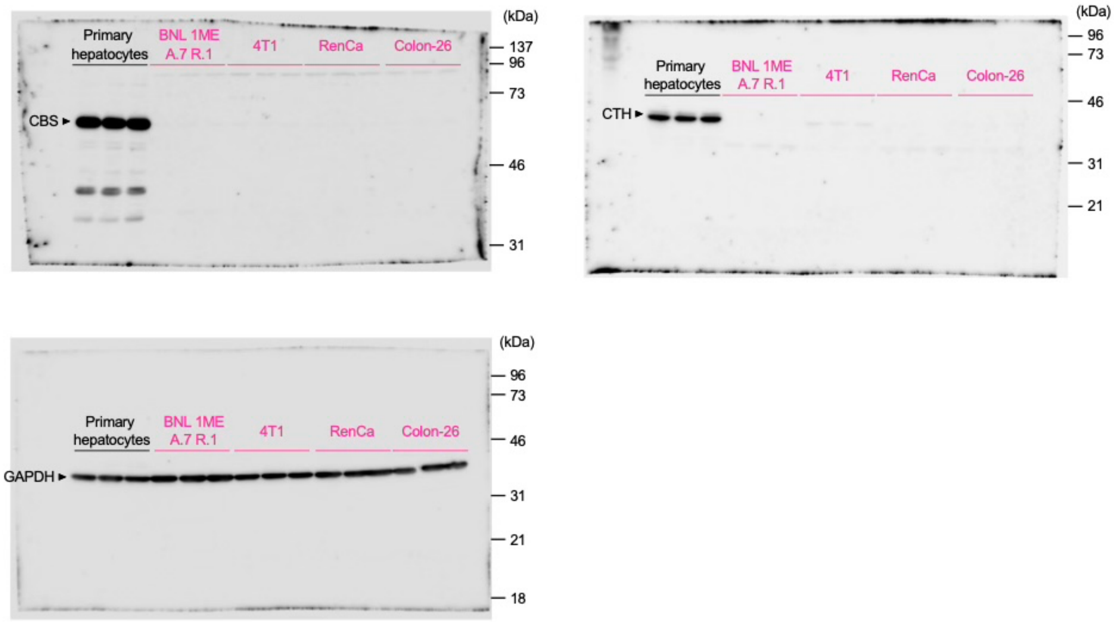

Supplementary Figure S27 Unedited full blots of Supplementary Figure S8B

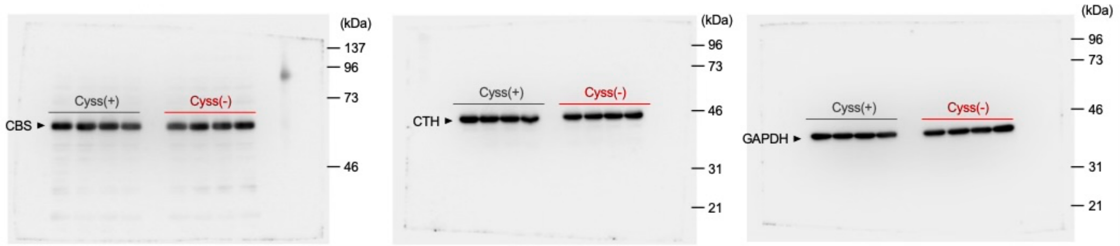

Supplementary Figure S28 Unedited full blots of Supplementary Figure S8C

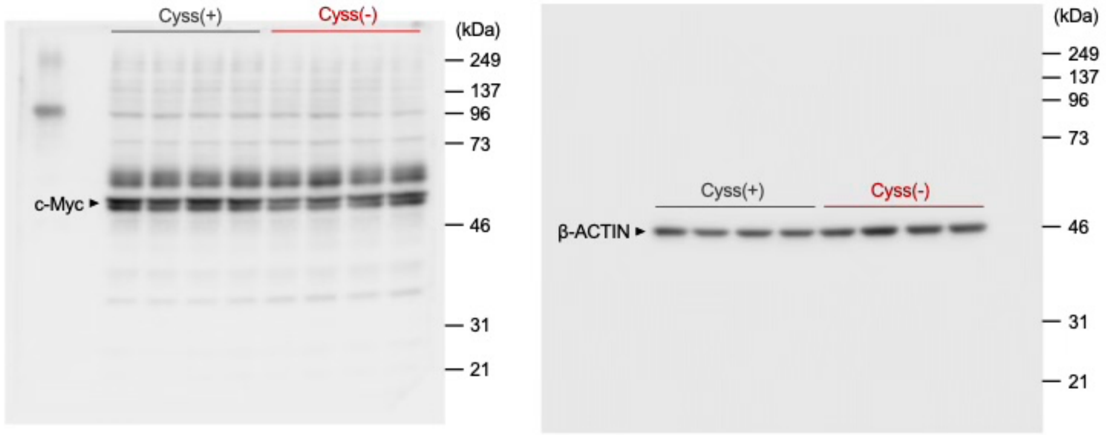

Supplementary Figure S29 Unedited full blots of Supplementary Figure S10

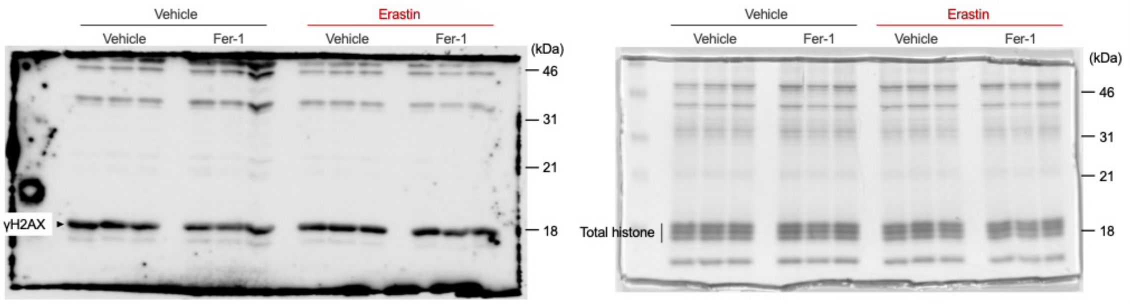

Supplementary Figure S30 Unedited full blots of Supplementary Figure S11D
